# Survival of the Curviest: Noise-Driven Selection for Synergistic Epistasis

**DOI:** 10.1371/journal.pgen.1006003

**Published:** 2016-04-28

**Authors:** Jon F. Wilkins, Peter T. McHale, Joshua Gervin, Arthur D. Lander

**Affiliations:** 1 Ronin Institute, Montclair, New Jersey, United States of America; 2 Center for Complex Biological Systems & Department of Developmental and Cell Biology, University of California, Irvine, Irvine, California, United States of America; Centre for Genomic Regulation, SPAIN; Georgia Institute of Technology, UNITED STATES

## Abstract

A major goal of human genetics is to elucidate the genetic architecture of human disease, with the goal of fueling improvements in diagnosis and the understanding of disease pathogenesis. The degree to which epistasis, or non-additive effects of risk alleles at different loci, accounts for common disease traits is hotly debated, in part because the conditions under which epistasis evolves are not well understood. Using both theory and evolutionary simulation, we show that the occurrence of common diseases (i.e. unfit phenotypes with frequencies on the order of 1%) can, under the right circumstances, be expected to be driven primarily by synergistic epistatic interactions. Conditions that are necessary, collectively, for this outcome include a strongly non-linear phenotypic landscape, strong (but not too strong) selection against the disease phenotype, and “noise” in the genotype-phenotype map that is both environmental (extrinsic, time-correlated) and developmental (intrinsic, uncorrelated) and, in both cases, neither too little nor too great. These results suggest ways in which geneticists might identify, a priori, those disease traits for which an “epistatic explanation” should be sought, and in the process better focus ongoing searches for risk alleles.

## Author Summary

The contribution of epistasis, or non-additive effects of risk alleles at different loci, to complex traits is much debated among human geneticists. In this study we use modeling and simulation to identify when evolutionary forces should drive epistasis to become a major part of the explanation for such traits. We simulate populations evolving in the presence of “phenotypic noise”, i.e. intrinsic and environmental variability in the relationship between genotype and phenotype, and focus specifically on traits that are substantially deleterious (e.g. fitness loss of at least 10%) and moderately common (population frequency between 0.1 and 10%). These criteria describe much “common”, heritable human disease, and knowing the expected contribution of epistasis to such diseases could greatly assist efforts to use genome-wide genetic associations to identify their mechanistic underpinnings. Here we show that, provided that appropriate levels and kinds of phenotypic noise are present during evolution, genetic contributions to disease traits can be expected to be strongly and synergistically epistatic, particularly among the most severely affected individuals in a population. By identifying scenarios most likely to select for synergistic epistasis, this study may help biologists identify when the extra effort is merited to search for combinatorial genetic interactions in human disease.

## Introduction

Patterns of inheritance suggest that genetic variation plays a central role in the etiology of common, serious diseases (e.g., autism, schizophrenia, multiple sclerosis, diabetes). Yet genetic variants found by Genome Wide Association Studies (GWAS) typically explain only a small proportion of the heritability of such disorders [1]. Some have proposed that causative alleles elude discovery because their effect sizes are too small, or because they are not included among the variants that are normally ascertained. Another proposed reason is epistasis, i.e. the combinatorial action of variants at different genetic loci, such that they contribute significantly to disease only when they appear together in an individual [2–4]. Epistasis, also referred to as gene-gene interaction, can be exceedingly difficult to detect in GWAS—due to computational as well as statistical challenges [5,6]—and much debate exists about the extent to which efforts should be made to search for it.

Epistasis is common in the experimental genetics of model organisms, but one cannot simply extrapolate the behaviors of the large-effect alleles favored by experimental geneticists to the small-effect alleles thought to dominate the landscape of standing genetic variation (the fact that a continuous function can be approximated as linear over a small enough interval implies that, as selection drives the effect sizes of alleles toward zero, non-linear interactions—i.e. epistasis—should become insignificant). Although quantitative-trait-locus mapping in populations of model organisms has verified the existence of substantial epistasis among two [7–10] and even three or more genes at a time [11], the number of replicated examples of strong epistasis underlying human traits—particularly disease traits—is modest [12,13]. Whether this paucity of examples reflects the methodological difficulties (e.g. lower statistical power) inherent in working with human populations, or the fact that the major genetic causes of disease lie elsewhere (e.g. among rare, large-effect variants) is one of the most hotly debated questions in human genetics.

Progress toward addressing this question could be enhanced if investigators knew when and where to look for epistatic interactions in the human genome. One tactic is to use existing mechanistic knowledge—e.g. known biochemical or genetic pathways associated with the physiology disrupted by disease—to narrow the search, thereby improving statistical power [6]. The drawback to this approach is that, by working only outward from what we know, we sacrifice much of the ability to detect anything radically new. An alternative approach is suggested by the fact that epistasis, when present, must have evolved, i.e. come into being via the usual forces of mutation, drift, and natural selection. If we could determine the circumstances under which diseases with a strong epistatic genetic component do and do not evolve, we might be able to use that information as additional prior knowledge in genetic association studies.

The evolution of epistasis has previously been investigated by several groups, who have reached a variety of conclusions about it, not all of which are mutually compatible [14–18]. The approach in the present study differs from that prior work in that it is focused not on the typical or average behaviors of populations, nor on traits in general, but rather on genetic diseases—by which we mean traits that should be under strong negative selection—and populations in which disease incidence is in the range of many common, serious human diseases, i.e. between 0.1% and 10%. Using both theoretical analysis and evolutionary simulation, we find that the extent to which epistasis explains a disease phenotype depends on the nature of the interaction between gene products (i.e. the biochemistry), the strength of selection on the trait, and especially on the nature and magnitude of stochastic and environmental fluctuations that influence gene function.

## Results

### Measures of Epistasis

To quantify epistasis is to capture the degree to which phenotypes cannot be accounted for by summing the phenotypic effects of variation at multiple genes, but one may approach this task in multiple ways. Among population geneticists, it is traditional to calculate the proportion of phenotypic variance, *σ*^2^, that can be explained by an additive model, i.e. $h_{pop}^{2}=\sigma_{A}^{2}/\sigma^{2}$, where $\sigma_{A}^{2}$ represents the maximum variance that a linear model can produce, as determined by linear regression (Section 1 in S1 Text). The quantity $h_{pop}^{2}$ is termed the additive, or narrow-sense, heritability, and if non-genetic contributions to variance can be neglected or corrected for (a situation that we denote by an asterisk in what follows), $1-h_{pop*}^{2}$ captures what is traditionally termed “statistical epistasis”.

Alternatively, if one assumes that a particular phenotype or set of phenotypes may be treated as reference (i.e. “wildtype”), one may measure epistasis from the standpoint of an individual. For example, if *Z(g)* represents the phenotype associated with a given genotype, *g*, then for a phenotype controlled by two loci, we may define

$$h_{ind*}(x,y|x_{0},y_{0})=\frac{\left[Z(x,y_{0})-Z(x_{0},y_{0})\right]+\left[Z(x_{0},y)-Z(x_{0},y_{0})\right]}{Z(x,y)-Z(x_{0},y_{0})},$$ (1)

where *h*_ind*_(x,*y|x*_*0*_,*y*_*0*_*)* represents the proportion of phenotypic difference between genotype *x*,*y* and reference genotype *x*_*0*_,*y*_*0*_ that is due to additive effects (extension to a greater number of loci is straightforward). The asterisk in (Eq 1) reminds us that this definition neglects non-genetic contributions to the phenotype, generalization to which will be introduced later. In this case, 1 − *h*_ind*_ measures what is often termed “functional epistasis”. Unlike statistical epistasis, functional epistasis may be further classified as synergistic (*h*_ind*_ < 1) or antagonistic (*h*_ind*_ > 1).

Although (Eq 1) is defined in terms of the genotype of a single individual, relative to a single reference, it is straightforward to generalize it to collections of “cases” and “controls”, as we in fact do later. Thus, the primary difference between functional and statistical epistasis is not that one is population-centered and the other is individual-centered, but rather that functional epistasis focuses on the causes of variation from a pre-specified “wild” or “normal” state, whereas statistical epistasis focuses on the structure of variation within a population overall. From the standpoint of understanding the molecular causes of human disease, or predicting who will develop disease, functional epistasis is the more relevant quantity, as foreknowledge of a “normal” phenotype, or range of phenotypes, is implicit in the notion of disease. It is thus the evolution of functional epistasis that we are most concerned with here.

**Fig 1:**
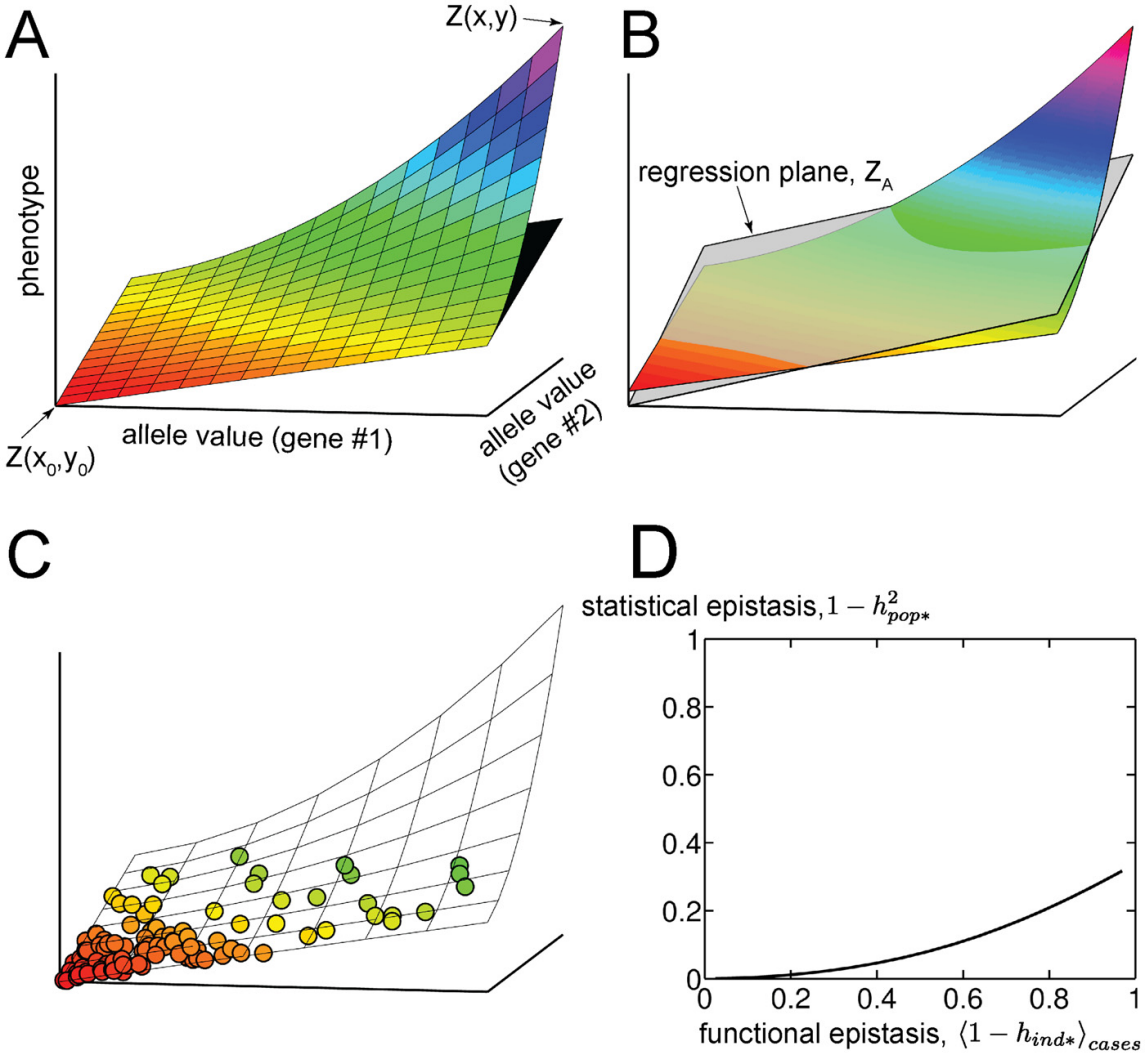
Epistasis at the individual versus population levels. (A) Functional epistasis. A phenotypic landscape (colored surface) is defined by Eq (S10) with ξ = 0.5 and *n* = 2. Allele values represent a quantitative measure of gene function given that the genotype is that allele. The difference between the phenotype value associated with two alleles (*Z(x*,*y)*), and the plane formed by the phenotype values of wildtype (*Z(x*_*0*_,*y*_*0*_*)*) and single-variant (*Z(x*_*0*_,*y) and Z(x*,*y*_*0*_*)*) genotypes (shown in black), provides a local measure of epistasis. (B) Statistical epistasis. Linear regression determines a best-fit plane (translucent surface; see Eq (S2)) that depends upon the distribution of genotypes in the population. (C) A sample from the population used to perform the regression in B; individuals (circles) are colored according to their phenotype values. The population distribution is that defined by Eq (S11), with *λ* = 0.2 (D) Low statistical epistasis can exist in the presence of high functional epistasis. Here functional epistasis (evaluated with respect to the genotype (*x*_0_,*y*_0_); see panel A) has been averaged over all case individuals, defined as the 1% of the population with the greatest phenotype values. The parameter ξ was varied while the other parameters were held fixed at *n* = 1, *λ* = 0.2, C = 1 (see Eq. (S10) and (S11)).

On the other hand, statistical epistasis is closely tied to the mechanics of evolution, because it is $h_{pop*}^{2}$ that effectively determines how natural selection acts on phenotypic variation [19]. This makes it relatively straightforward to develop insights about how statistical epistasis evolves, but such insights may be unhelpful with regard to functional epistasis, because the latter can exist in the absence, or near absence, of the former, e.g. [14,20–22]. How this can happen is shown in Fig 1.

Fig 1A depicts an arbitrary two-dimensional phenotypic landscape (i.e. allele values for each of two genes are on the independent axes, and phenotype value is on the dependent axis) and illustrates how *h*_ind*_(x,*y|x*_*0*_,*y*_*0*_*)* simply reflects the nearness of *Z(x*,*y)* (top-most point of colored landscape) to the plane formed by *Z(x*_*0*_,*y*_*0*_*)*, *Z(x*_*0*_,*y)* and *Z(x*,*y*_*0*_*)* (black surface visible directly below *Z(x*,*y)*). On the other hand, $h_{pop*}^{2}$ reflects how well the landscape is fit by a plane (translucent surface in Fig 1B), where the fit is weighted by the population distribution (a sample from one possible population distribution is shown in Fig 1C). In order for any sort of epistasis to occur, the landscape must be non-linear, but a large discrepancy between *h*_ind*_ and $h_{pop*}^{2}$ can occur at genotypes where the non-linearity is strong while the population density is low.

For example, it is relatively easy to construct scenarios in which nearly all of the phenotypic variation displayed by “diseased” individuals is explained by functional epistasis, even though there is little statistical epistasis in the population as a whole (Fig 1D). Even if diseased individuals represent as much as 50% of a study population (as in the artificially selected populations of “cases” and “controls” that are assembled for GWAS), statistical epistasis can still be well below the level of functional epistasis of the cases, relative to the controls (S1A and S1B Fig; section 2 in S1 Text). In our evolutionary simulations, described below, we typically choose a phenotypic threshold between health and disease that assigns ~1% of the population to the latter category, consistent with the ascertainment level of many “common” human disorders. We thus focus on the questions of when, in the course of evolution, situations should arise in which functional epistasis is large among a subset of individuals of this magnitude.

### Epistasis Is Selected against in the Simple LP Model

**Fig 2:**
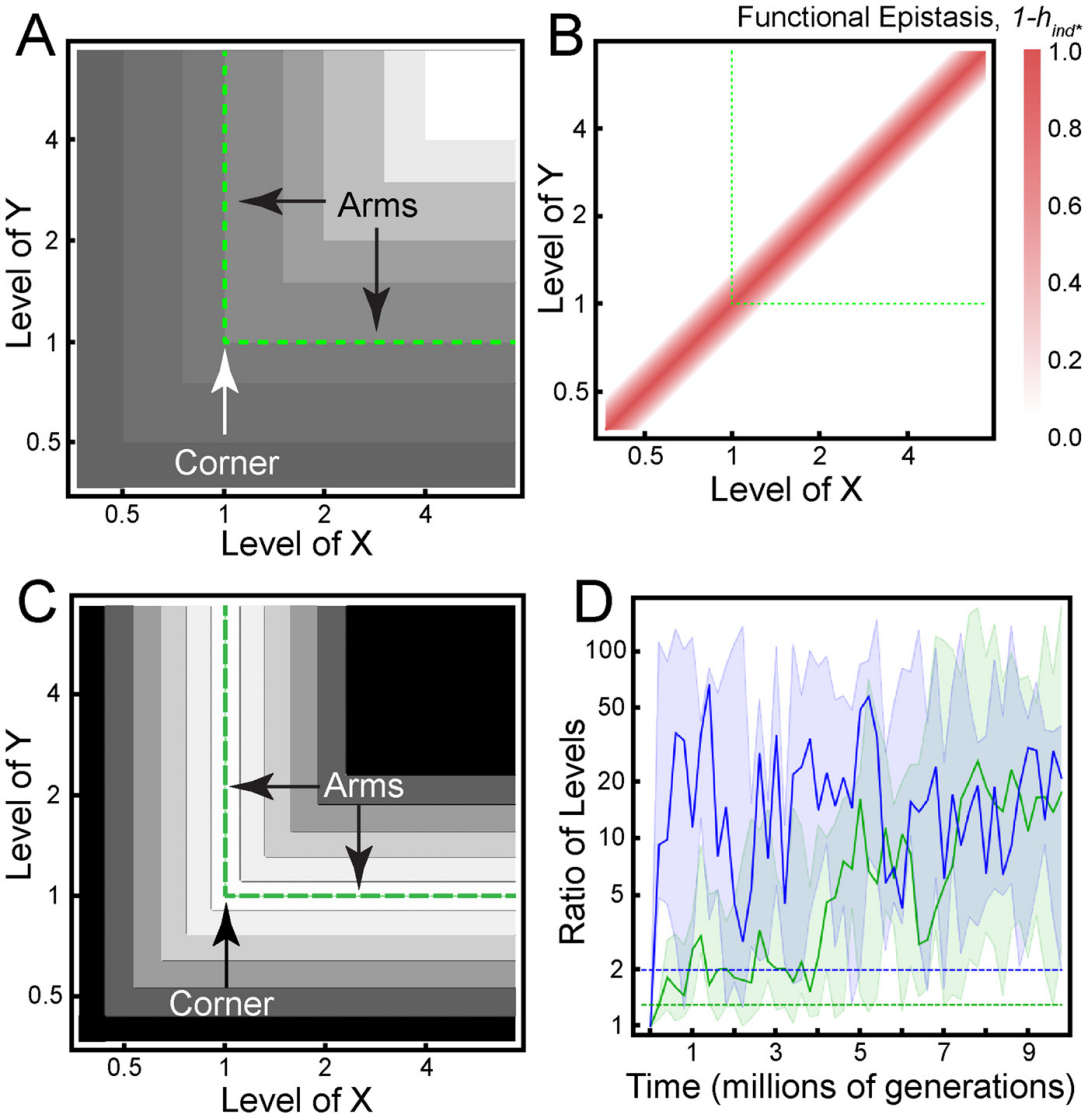
Epistasis does not evolve in the simple LP model. (A) Phenotype value as a function of input values in the simple LP model, *z* = Min[*x*,*y*]. Lighter shading represents higher phenotype values. (B) At each combination of *x* and *y*, we calculated the contribution of epistasis to the change in phenotype resulting from a 30% increase in each input value. Near the diagonal, the "sign" of local functional epistasis depends on whether mutations increase or decrease *x* and/or *y*. Mutations that increase (decrease) both *x* and *y* result in synergistic (antagonistic) epistasis. The nature of epistasis between a mutation that increases one input value and a mutation that decreases the other will depend on the precise values of *x* and *y*. (C) Fitness landscape given by (Eq 2), with the optimal value of *z* being *z*_*opt*_ = 1 and the strength of selection being *s* = 1. Lighter shading represents higher fitness. (D) Simulated time course of a population evolving along the optimal-fitness ridge indicated by the green dashed lines in C. The population-mean input values (i.e. average of individual (*x*,*y*) values weighted by their corresponding frequencies in the population) were calculated every 200,000 generations and their ratio (*y/x* if *y*>*x*; *x/y* if *x*>*y*) recorded on the vertical axis, which indicates how far the population center of mass is from the corner of the ridge. The green band and curve indicate the minimum, maximum, and median values from five independent simulations with an average mutational effect size of *σ*_μ_ = 0.1. The blue band and curve correspond to a larger mutational effect size (*σ*_μ_ = 1.0). The region around the corner over which we expect to observe functional epistasis is limited by the effect size of a typical mutation (indicated by the horizontal dashed lines drawn at a distance of *σ*_μ_ from the corner). Initially, all individuals in the population lie at the corner (*x* = *y* = 1).

We began by considering a particularly simple model of non-additive gene-gene interaction—the Limiting Pathway (LP) model—that can be applied to a variety of biological processes [23]. In this model, a trait with value *z* depends on the rate-limiting value of a number of genetically controlled inputs, e.g., in the case when the number of inputs is two, *z* = Min[*x*, *y*], where *x* and *y* represent the inputs from two genes (Fig 2A). These might represent the expression levels of two different polypeptides that combine to form a multi-subunit protein, the abundance of which is given by *z*. Alternatively, *x* and *y* could represent rates of synthesis of small molecules, such as enzyme cofactors, that catalyze a process occurring at rate *z*. For systems involving more than two inputs, we have *z* = Min[*x*_*1*_, *x*_*2*_, *x*_*3*_…], but for simplicity we will focus here on the two-component model.

We assume there is an optimal phenotype value, *z*_*opt*_, and that the trait quantified by *z* is under stabilizing selection, so that fitness is reduced when *z* falls above or below *z*_*opt*_. Thus, at evolutionary equilibrium, we expect the wild-type levels of the gene products, which we will call *x*_0_ and *y*_0_, to be such that Min[*x*_0_, *y*_0_] = *z*_*opt*_. The representation of this condition on the phenotypic landscape, i.e. the intersection of the plane *z* = *z*_*opt*_ with the function *z* = Min[*x*, *y*], defines two perpendicular “arms”, meeting at a “corner” (green dashed lines in Fig 2A).

Only when populations reside near this corner, *i*.*e*., *x*_0_ ≈ *y*_0_, can significant epistasis occur. For example, under such conditions mutations that increase *x* or *y* may have little phenotypic effect individually, but a large effect in combination (synergistic epistasis). In contrast, for a population residing on one of the arms—e.g., *x*_0_>>*y*_0_, corresponding to the horizontal arm in Fig 2A—mutations that increase or modestly decrease *x* will have no phenotypic effect; mutations that affect *y* will alter *z* proportionately; and the combined effect of both mutations will be no different from the sum of their individual effects.

For any pair of wild-type input values (*x*_0_, *y*_0_) and mutation effect sizes (Δ*x*, Δ*y*), we may quantify the magnitude of functional epistasis as 1 –*h*_ind*_(*x*_0_ + Δ*x*_0_,*y*_0_ + Δ*y*_0_ | *x*_0_,*y*_0_), according to (Eq 1), as described above. For a given mutation effect (Δ*x* = Δ*y* > 0), we repeat this calculation for different combinations of wild-type values *x*_0_ and *y*_0_, to produce an epistasis map (Fig 2B). Asking whether epistasis will tend to evolve in the LP model thus amounts to asking whether input values in natural populations will evolve towards and remain at the corner in Fig 2A. Re-plotting Fig 2A in terms of fitness, which we take to be the following generic function of phenotype value

$$w(z)=\text{exp}\left(-s{\left(\text{ln}\frac{z}{z_{opt}}\right)}^{2}\right),$$ (2)

(where the strength of stabilizing selection is quantified by *s*) yields an L-shaped ridge on which any point along the ridge is equally and maximally fit (Fig 2C). Given this fitness landscape, our naïve expectation might be that, under selection and drift, populations should follow a random walk along the ridge, with individual input values equally likely to be clustered around any ridge location.

However, evolutionary simulations (see Methods) show that selection drives populations away from the corner and out onto the arms of the ridge (Fig 2D). To understand why, note that at “arm” locations where *y << x*, mutations affecting *x* will be selectively neutral. But if the population is close to the corner, such that *y < x < y + σ*_μ_, where *σ*_μ_ is the typical mutational step size, mutations that reduce *x* will be deleterious. Because of the increased frequency of deleterious mutations near the corner, natural selection disfavors genotypes with input values within *σ*_μ_ of the corner (compare shaded regions at long times with horizontal dashed lines in Fig 2D). It follows that the population is least likely to be found precisely where epistasis is strongest.

### A More Biologically Realistic LP Model

Is the evolutionary instability of epistasis in the LP model something common to all genotype-phenotype relationships in which strong synergistic epistasis can arise? Or is the LP model, as currently formulated, insufficiently general? To approach this question, we explored two modifications of the LP model.

First, we generalized the phenotype function with the aid of parameter *k*, which allows us to vary the strength of interaction between pathways:

$$z=\frac{1}{2}\left(k+x+y-\sqrt{{\left(k+x+y\right)}^{2}-4xy}\right)$$ (3)

**Fig 3:**
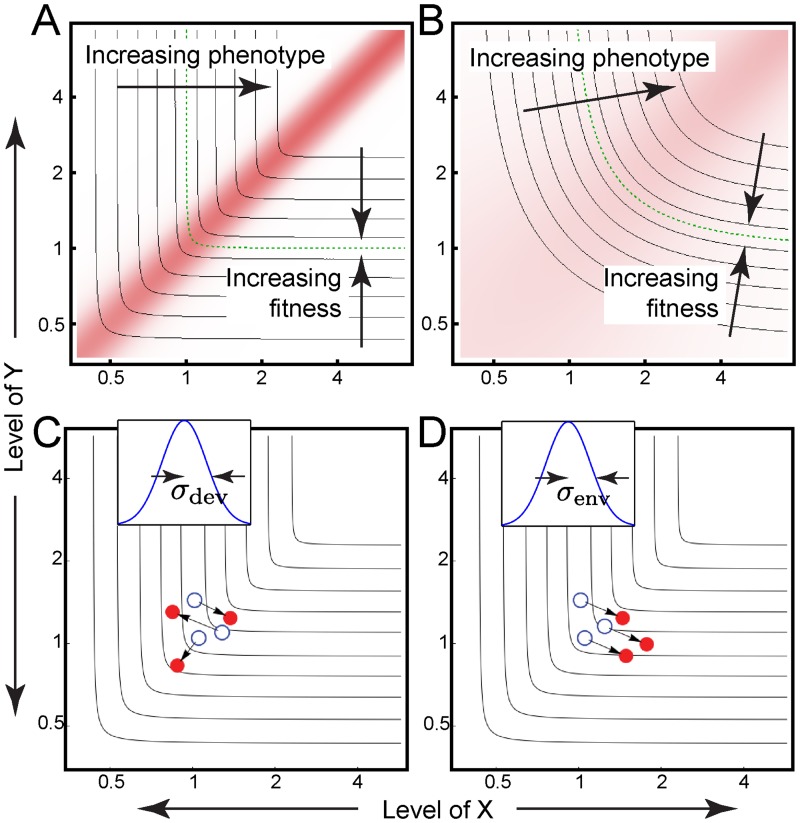
The generalized LP model. (A, B) Epistasis landscape (red shading) and common contours of the phenotype and fitness landscapes (black lines) for *k* = 0.005 (A) and 0.5 (B). (C, D) Schematic illustrating how phenotypic noise can be viewed as random perturbations of the input values, transforming nominal values (white) into effective values (red). The magnitudes of the perturbations are drawn from a Gaussian distribution defined on the natural logarithm of gene levels (insets). This sampling process is repeated for each individual independently (developmental noise; C) or performed once for all individuals (environmental noise; D); see Methods.

When *k* = 0, (Eq 3) reduces to the simplified LP model, *i*.*e*. *z* = Min[*x*,*y*]. If *x* and *y* were to represent the concentrations of two reactants, and *z* the concentration of their product, then *k* would represent the corresponding biochemical dissociation constant. In this interpretation, if we choose our units so that *z*_*opt*_ = 1, *k* >> 1 would correspond to weak binding while *k* << 1 would be tight binding. We may also interpret *x* and *y* in (Eq 3) as rates of synthesis of gene products, though the physiological meaning of *k* would then be different, incorporating binding and rates of degradation. As shown in Fig 3A and 3B, the effect of increasing *k* is to make the corner in the fitness landscape more round. This increases (for any given mutational step size) the range of values of *x* and *y* over which significant epistasis will occur, but also decreases the maximum potential magnitude of that epistasis.

Second, we added phenotypic noise into the model. By noise we mean processes that add randomness into the genotype-phenotype relationship. For purposes of this analysis, we divide noise into two categories: developmental and environmental.

By developmental noise, we mean processes that affect each individual in the population independently and are not substantially correlated between parents and offspring (Fig 3C). An example of developmental noise would be idiosyncratic variation in the biochemical processes underlying development, such that individuals with identical genotypes do not necessarily display identical phenotypes. Other factors that could contribute to developmental noise by this definition would include micro-environmental effects that vary from individual to individual and genetic variation at loci not explicitly incorporated into the model (assuming high recombination).

By environmental noise, we mean processes that have a coordinated effect on all individuals in a population, and may persist from one generation to the next (Fig 3D). Processes like climate change, dietary change, and cultural change, which have the potential to fluctuate on time scales of many generations, would fall into this category. Although it is common to model such processes as perturbations to the fitness (i.e., “fluctuating selection”, e.g., [24]), we model these processes as perturbations to phenotype, in order to facilitate comparison between the two classes of noise.

Accordingly, we take noise generally to be a process that transforms an individual’s *nominal* input values (the values of *x* and *y* dictated by the individual’s genotype; white circles in Fig 3C and 3D; S2A and S2C Fig) into an *effective* set of input values (red circles in Fig 3C and 3D; S2B and S2D Fig) that produce, via (Eq 3), an altered phenotype. The effective input values can be thought of as the set of values that would produce the observed phenotype in the absence of any noise.

Motivated by the fact that cell-to-cell variation in the levels of gene products is often found to fit log-normal distributions [25,26], we implement developmental noise in *x*, say, via the transformation

$$\text{ln}x_{e}=\text{ln}x+\Delta x$$ (4)

where Δ*x* is drawn from a normal distribution with mean zero and variance *σ*_*dev*_^2^ (Fig 3C; inset). Perturbations to *y* are performed independently using the analogous transformation.

With environmental noise, the same perturbation affects the *x*- and *y*-values of all individuals in the population (Fig 3D; inset). Although that perturbation is also drawn randomly (this time from a zero-mean Gaussian with standard deviation σ_*env*_), it is not re-drawn at random every generation, as environmental effects may vary on slow time scales. Details of how this is modeled are provided in the Methods section, but for all results presented in the main text the Δ*x* and Δ*y* values for environmental noise have an autocorrelation time of 27 generations.

### Noise Stabilizes the Corner

How should we expect phenotypic noise to affect population dynamics on the generalized LP landscape? So long as the autocorrelation time of phenotypic noise is short relative to the timescale on which natural selection drives genetic change, we can approximate the effect of natural selection on a particular combination of input values by averaging over their different fitness realizations due to noise. The resulting “effective fitness” measures how robust the phenotype (and nominal fitness) of that particular set of input values is to random variation of their values.

**Fig 4:**
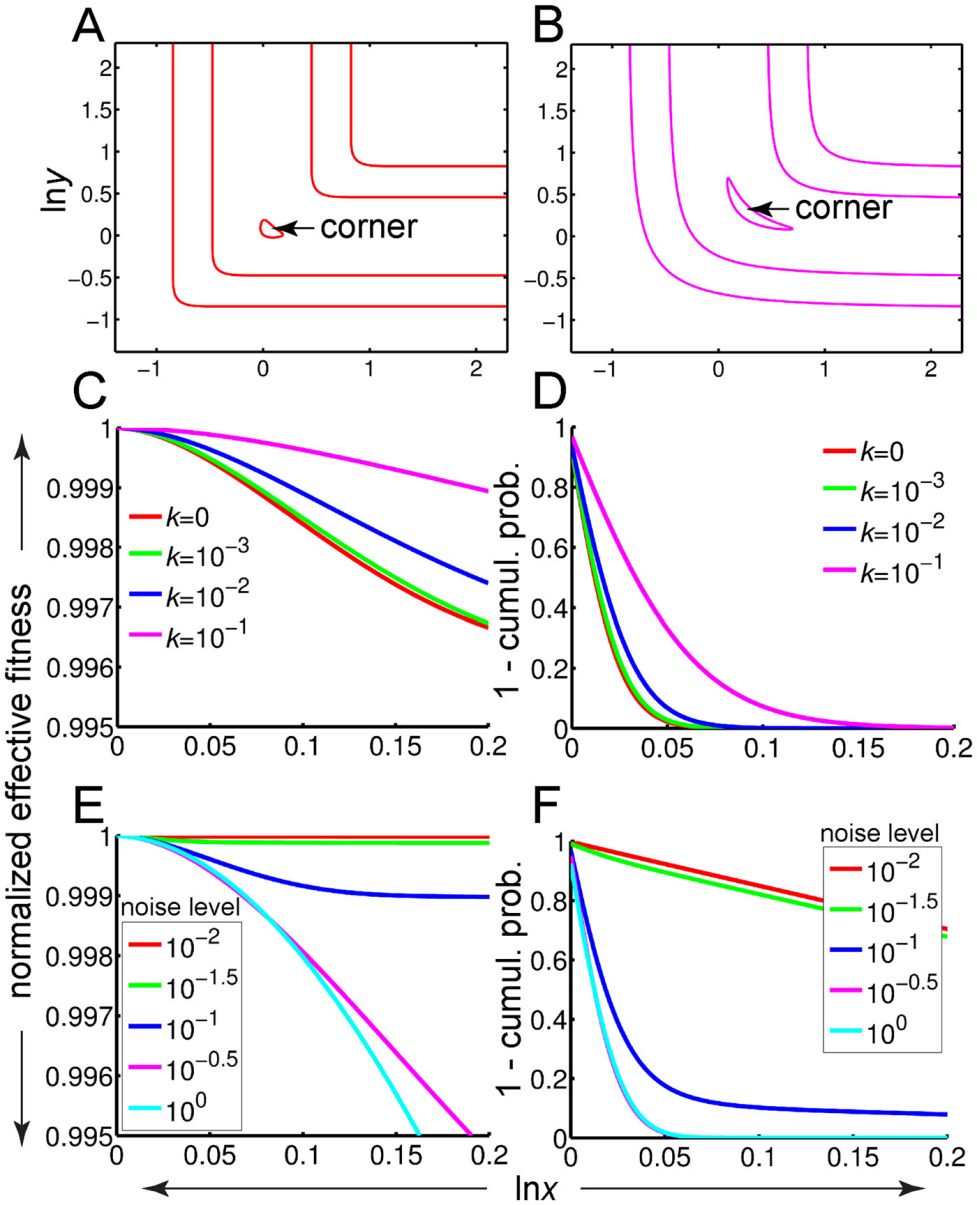
Phenotypic noise stabilizes the corner in the random-walk approximation. (A, B) Contours of the effective fitness landscape, *w*_*e*_(*x*,*y*), for *k* = 0 (A) and *k* = 0.1 (B). Effective fitness resulting from phenotypic noise is computed at any given position by averaging neighboring fitness values using a rotationally symmetric normal distribution (on log-transformed input values) with a standard deviation (“noise level”) of 0.2. Contour lines correspond to *w*_*e*_ = 0.5, 0.8, 0.99. (C) Normalized effective fitness profiles, *w*_*e*_(*x’*)/*w*_*e*_(0), along the pseudo-horizontal arm of the optimal fitness ridge, projected onto the *x*-axis and shifted horizontally so as to align the corners. *x’* = ln*x*. (D) Probabilities that the population is found at least the indicated distances from the corner. (E, F) Same as panels C and D, respectively, except now the noise level is changed while the interaction constant is kept fixed at *k* = 10^−4^. Throughout, population size is *N* = 5000, optimal phenotypic value is *z*_*opt*_ = 1 and selection factor is *s* = 1.

When we compute this effective fitness landscape, we find a narrow peak at the corner for *k* = 0 (Fig 4A) that develops into a broad peak at *k* = 0.1 (Fig 4B). Though a peak at the corner is intuitively expected to localize the population there, it is less clear how the *shape* of the peak affects localization. Since the motion of the population is driven by fitness differences, a narrow peak (and a steep fitness gradient) should exert a strong force driving the population towards the corner. Yet that force can only be felt in the immediate vicinity of the corner, potentially allowing populations located elsewhere to drift away from the corner. On the other hand, though a broad peak exerts a weaker force (due to its shallower fitness gradient), its range of influence is greater, potentially drawing populations to the corner that would have otherwise escaped it.

To find out which landscape best stabilizes the corner at steady state, we mapped the evolution of a population on the 2D effective fitness landscape to a 1D random walk problem (Section 3 of S1 Text). The random-walk theory predicts that localization at the corner depends monotonically on *k*, with the greatest degree of corner localization expected at the smallest values of *k* (Fig 4D)—precisely the conditions under which epistasis is strongest (Fig 3A). Assuming that *k* is low enough, we next asked how corner localization depends on the noise level, by which we mean the width of the Gaussian from which the phenotypic perturbation is drawn (*σ*_*dev*_ or *σ*_*env*_; Fig 3C and 3D). When the noise level drops below 0.1, the random walk is effectively unbiased, with the effective fitness profile being approximately flat (Fig 4E) and the probability of finding the walker at least a given distance from the corner falling linearly with distance (Fig 4F).

**Fig 5:**
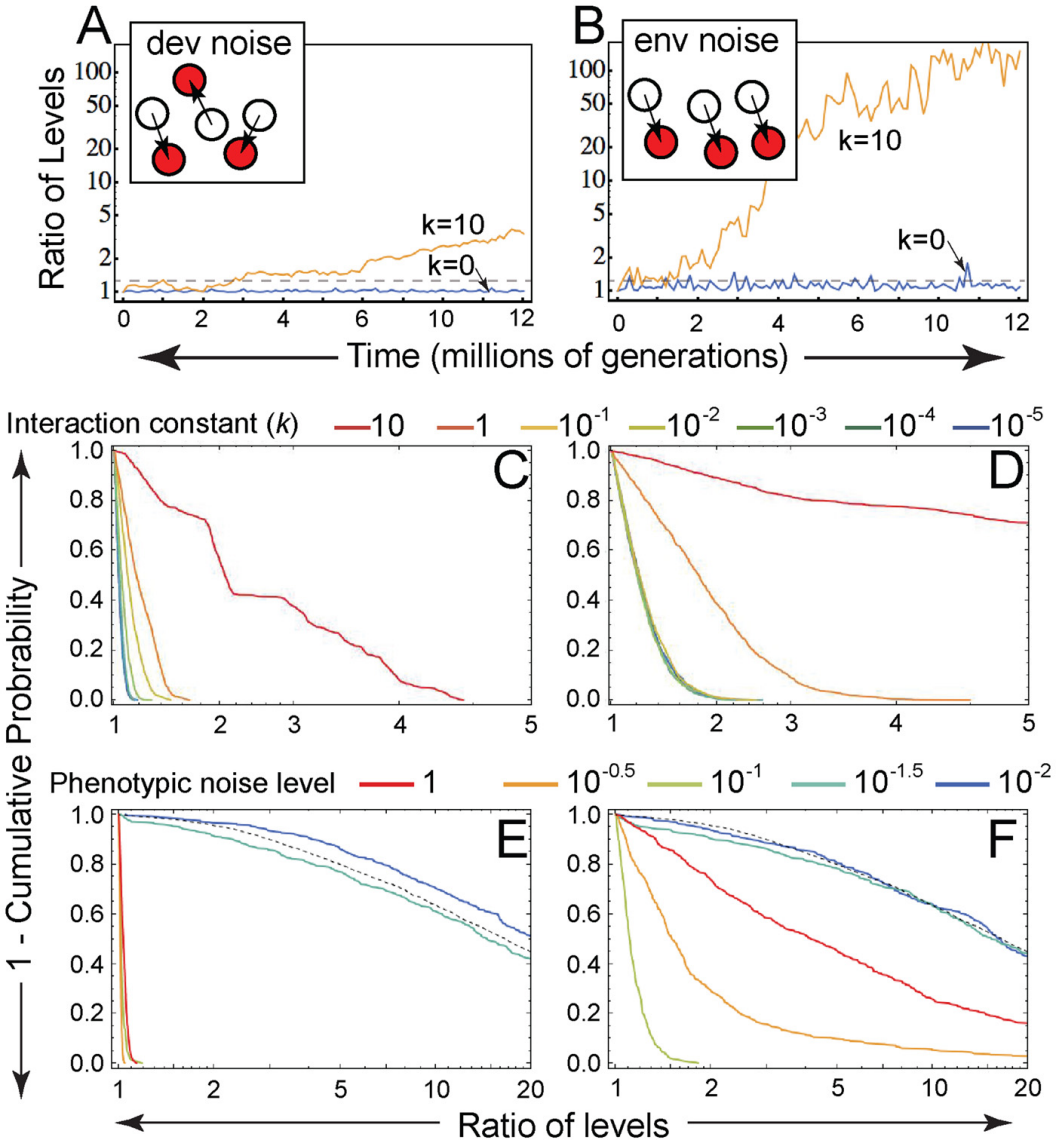
How the character of phenotypic noise affects corner stabilization in simulations. (A, B) The full evolutionary model (see Methods) was used to simulate time courses of the population center of mass (solid lines) for two different values of the interaction constant, *k*, subject to either pure developmental noise (A) or pure environmental noise (B). The proximity of the corner within which epistasis is expected is indicated by the horizontal dashed lines drawn at a distance of *σ*_μ_ = 0.1 from the corner. (C–F) The probability that the population is at least a given distance from the corner for various values of *k* (C, D), and various levels of developmental and environmental noise, σ_*dev*_ and σ_*env*_, respectively (E, F). In A and C, the phenotypic noise level is *σ*_*dev*_ = 0.1; in B and D it is *σ*_*env*_ = 0.1. In E and F, *k* = 10^−4^, and the black dashed line is the limiting case of no phenotypic noise (σ_*dev*_ = σ_*env*_ = 0). Mutational step size in C–F was *σ*_μ_ = 1. Throughout, data in the left and right columns correspond to purely developmental and purely environmental phenotypic noise, respectively.

The random-walk theory, though providing significant insight, is an approximation that is expected to hold only when the product of the population size and the mutation rate is small, i.e. *Nμ* << 1. To address more general conditions, we examined the population dynamics using evolutionary simulations. Time courses plotted from individual simulations show that both developmental and environmental noise are effective at localizing the population in proximity of the corner (where epistasis can arise) when the interaction parameter, *k*, is small enough (Fig 5A and 5B; see also S2E and S2G Fig). To summarize the degree of corner localization under a given set of parameter values, we sampled simulations at intervals of 2*N* generations (where *N* is population size) and computed the fraction of simulations where the population is at least a given distance from the corner (Fig 5C and 5D). For both developmental and environmental noise, simulated data confirm the general trend predicted by the random-walk theory: the sharper the corner (and the greater the potential for strong epistasis), the more effective phenotypic noise is at localizing the population there (see also S4 Fig).

We repeated the evolutionary simulations for a range of noise levels, either pure developmental or pure environmental. Fig 5E shows that the dependence of corner localization upon developmental noise is remarkably similar to that predicted by the random-walk theory (Fig 4F). However, when the phenotypic noise is environmental, the results of theory and simulation diverge. Evolutionary simulations show that corner localization depends non-monotonically on environmental noise level such that only intermediate noise levels (*σ*_*env*_ ≈ 0.1) hold the population near the corner (Fig 5F). In contrast, the theoretical dependence on noise level was always monotonic (Fig 4F).

The accuracy of random-walk theory in predicting the role of developmental but not environmental noise in simulations reflects the fact that the theory takes as input an *effective* fitness landscape representing the average effect of phenotypic noise. While such averaging is expected to faithfully capture pure developmental noise, which is independently realized for each individual and at each generation (Fig 3C), this approximation does not account for the correlations (among individuals and across generations) intrinsic to our implementation of environmental noise (Fig 3D).

In summary, both theory and simulation show that phenotypic noise drives populations to the corner of the generalized LP fitness landscape, with the greatest degree of corner localization expected at the smallest values of *k*—the same condition under which the genotype-phenotype map is most nonlinear (Fig 3A). Though such nonlinearity is clearly a necessary condition for generating epistasis in individuals of the population, it is certainly not sufficient. To shed light on human disease biology, we next engaged the full power of our simulations to determine the conditions under which individuals affected by disease exhibit epistasis relative to healthy controls, and the frequencies at which such individuals may arise.

### Expectations for Epistasis in Case-Control Studies

**Fig 6:**
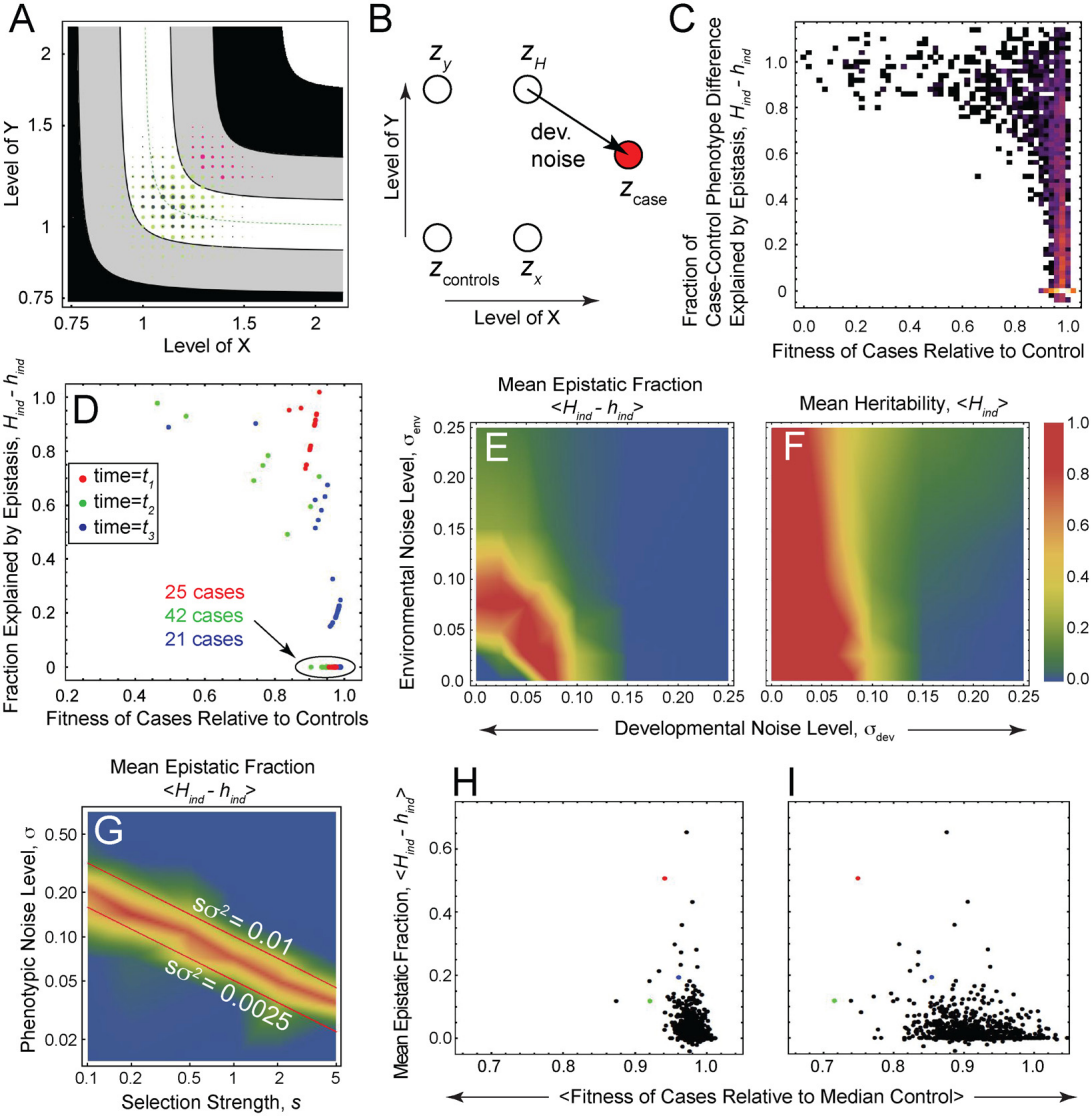
When to expect epistasis in case-control studies. (A) Distribution of *effective* input values among all individuals (green) in a simulated population and conditioned upon individuals being “cases” (pink) or “controls” (dark blue). Cases consist of the 1% of the population with the highest phenotype values; 500 controls are sampled from the 50% of the population whose phenotype values lie between the 25^th^ and 75^th^ percentiles. The area of each circle is proportional to the number of individuals whose effective input values fall within a square bin at the circle’s location. Other parameter values are *z*_*opt*_ = *s* = 1; *N* = 5000; *k* = 0.01; *σ*_μ_ = 0.1; *σ*_*dev*_ = 0.1; *σ*_*env*_ = 0. (B) Illustration showing how phenotype differences between cases and controls are partitioned into heritable (additive plus epistatic) and non-heritable components (see main text). *z*_*controls*_ represents the phenotype of a hypothetical control whose position is given by the median of the effective input values (i.e. including developmental noise) of all actual controls. (C) Joint distribution of the epistatic fraction, *H*_*ind*_*—h*_*ind*_, and fitness (relative to mean control fitness) over all cases (top 1% of phenotypes) and all sampled time points. The color of each pixel reflects the number of cases with the corresponding fitness and epistasis values. Parameter values are *k* = 0 and *σ*_*dev*_ = *σ*_*env*_ = 0.05. (D) Joint distribution of epistasis and fitness of cases (top 0.5% of phenotypes) for each of three time points, *t*_*1*_, *t*_*2*_, *t*_*3*_, distinguished by color. Parameter values are *σ*_*dev*_ = *σ*_*env*_ = 0.05. (E—G) Heat maps with intensity representing epistatic fraction, *H*_*ind*_*—h*_*ind*_ (E, G) and heritability, *H*_*ind*_ (F) averaged over all cases with relative fitness < 0.9 and all sampled time points. In G, the *y*-axis indicates the total noise level $\sigma =\sqrt{\sigma_{dev}^{2}+\text{​}\sigma_{env}^{2}}$, with *σ*_*dev*_ = *σ*_*env*_, and *σ*_μ_ = 1. (H) Distribution over all sampled time points of epistasis and fitness (each averaged over all cases at the corresponding time point). Colored data points correspond to the three time points sampled in panel D. (I) Same as H but, at each sampled time point, case phenotype and case fitness were assessed immediately after an abrupt 5-fold increase in selection strength from *s* = 1 to *s* = 5. The corresponding fold change in relative fitness of each case is approximately *w*^(*s*'/*s*)-1^ (which follows from (Eq 2)), where *w* is the relative case fitness prior to the selection shift. See also S12 Fig. Parameter values in H and I were *k* = 0; *σ*_μ_ = 1; *σ*_*dev*_ = *σ*_*env*_ = 0.05.

We modeled case-control studies of common disease (prevalence ≈ 1%) by sampling individuals with extreme and typical phenotype values (Fig 6A; S2 Fig), which we denoted “cases” and “controls”, respectively. For each case individual, we partitioned the difference between its phenotype value, *z*_*case*_, and the phenotype associated with the median input values of the control samples, *z*_*controls*_, into a fraction corresponding to the difference in nominal input values,

$$H_{ind}=\frac{z_{H}-z_{controls}}{z_{case}-z_{controls}},$$ (5)

which we refer to as the “heritability”, and a non-heritable fraction, 1-*H*_*ind*_. The quantity *z*_*H*_ represents the heritable phenotype of the case individual, which we approximated by its phenotype value before *developmental* noise was added (Fig 6B). In contrast, *environmental* noise is not expected to affect phenotype heritability assessed either within a generation (since genetically identical individuals have the same phenotype even after the addition of environmental noise) or between two consecutive generations (since environmental noise persists over many generations). Note that *H*_*ind*_, closely resembles the concept of broad-sense heritability as used by human geneticists, but is defined here at the level of a single individual, rather than the population as a whole. We can naturally partition *H*_*ind*_ into an additive fraction,

$$h_{ind}=\frac{\left(z_{x}-z_{controls})+(z_{y}-z_{controls}\right)}{z_{case}-z_{controls}},$$ (6)

and an “epistatic fraction”, *H*_*ind*_—*h*_*ind*_, where *z*_*x*_ and *z*_*y*_ represent the phenotype values of the single mutants defined by the heritable input values of the case individual (Fig 6B).

Epistatic fraction is a generalized measure of functional epistasis that is valid in the presence of phenotypic noise. To see this, note that, in the absence of noise, *H*_*ind*_ reduces to *1* and *h*_*ind*_ reduces to *h*_ind*_(*x*,*y* | *x*_0_,*y*_0_), where *h*_ind*_ is defined by (Eq 1) and the input values (*x*, *y*, etc) are determined by the relations *Z(x*,*y) = z*_*H*_ = z_*case*_ and *Z(x*_*0*_,*y*_*0*_*) = z*_*controls*_. Thus *H*_*ind*_—*h*_*ind*_ reduces to *1*—*h*_ind*_, which is the definition of functional epistasis presented earlier. The no-noise limit is instructive for another reason: it turns out that *h*_ind*_ evaluated at one population standard deviation relative to the median control sample directly determines the narrow-sense heritability used in human genetics, $h_{pop*}^{2}$ (S1C Fig; see also Section 1 in S1 Text).

We measured the epistatic fraction of case-control phenotype difference, *H*_*ind*_*—h*_*ind*_, and the fitness (given by (Eq 2), normalized by the mean fitness of the control samples) for each case individual and for each time point sampled during the course of our evolutionary simulations. Fig 6C (data aggregated over all time points) and 6D (data stratified by time point) both show that, though the vast majority of individual case phenotypes are largely additive (*H*_*ind*_ ≈ *h*_*ind*_), significantly unfit case individuals (i.e. fitness < 0.9) are *always* predominantly epistatic (*H*_*ind*_*—h*_*ind*_ *>* 0.5) with respect to controls. Put another way, although severe disease may arise infrequently in these simulations, when it does, the explanation for it is generally epistatic.

Because these evolutionary simulations keep track of individual mutations across generations, we can also calculate the frequency, in simulated populations, of the individual alleles that account for the epistatic interactions that give rise to unfit cases. As S5 Fig. shows, alleles involved in producing exceedingly unfit individuals (fitness < 0.75) tend to be relatively rare (frequency between 0.2% and 0.5%), whereas in somewhat less severely unfit cases (fitness between 0.75 and 0.9), causal alleles may display frequencies >1%, in the range of frequencies associated with minor alleles that can be followed in GWAS.

Fig 6E shows how the epistatic fraction, *H*_*ind*_*-h*_*ind*_, averaged over all substantially unfit cases (those with relative fitness < 0.9), varies with changing levels of phenotypic noise. In the absence of developmental noise (vertical axis of Fig 6E), strong, synergistic epistasis (<*H*_*ind*_*-h*_*ind*_> > 0.5) is observed only when environmental noise is of intermediate strength, because only then is the population localized to the corner (Fig 5F). More surprising, at first sight, is the fact that, in the absence of environmental noise (horizontal axis of Fig 6E), developmental noise must also be of intermediate strength, and is even more constrained than environmental noise (compare the extent of the red region along the two cardinal axes in Fig 6E). This constraint on developmental noise level originates in a trade-off between its effects on corner localization and (total) heritability, *H*_*ind*_. In the absence of environmental noise, modest developmental noise localizes the population to the corner (Fig 5E), favoring epistasis (σ_*dev*_<0.075; Fig 6E); on the other hand, large-amplitude developmental noise kills heritability (Fig 6F; S2A Fig), and with it, epistasis (σ_*dev*_>0.075; Fig 6E), which is bounded above by heritability (recall that heritability, *H*_*ind*_, is partitioned into an additive portion, *h*_*ind*_, and an epistatic portion, *H*_*ind*_*-h*_*ind*_). Thus, there is a range of developmental noise levels (σ_*dev*_≈0.05–0.10; Fig 6E) capable of localizing the population near the corner while preserving heritability.

How does the degree of epistasis among unfit (fitness <0.9) cases depend upon the other key ingredient of the extended LP model—the interaction parameter, *k*? While decreasing *k* below 0.01 does not affect corner stability (S4 Fig), it does increase the mean epistatic fraction, <*H*_*ind*_*-h*_*ind*_> significantly (S6 Fig; also compare Fig 3A with 3B). Thus, by making the LP landscape as nonlinear as possible, by sending *k* to zero, one maximizes the strength of epistasis one expects to observe in unfit case individuals.

The fact that the relative fitness of a case individual is such a strong predictor of its probability of being epistatic (Fig 6C and 6D and S7 Fig) prompted us to ask how epistasis is affected by the degree to which a trait is under selection, as measured by the selection factor, *s*, in (Eq 2). Fig 6G shows that the trait of interest must be under a certain amount of selection before we may expect strong epistasis among severely affected cases. That level of selection varies inversely with the level of phenotypic noise, $\sigma =\sqrt{\sigma_{dev}^{2}+\text{​}\sigma_{env}^{2}}$, as indicated by the red band in Fig 6G. In fact, this figure shows that epistasis depends primarily on the product *sσ*^*2*^ (see also Section 4 in SI), so that increasing the strength of selection is equivalent to increasing the noise variance. Epistasis is greatest for intermediate values of *sσ*^*2*^ (in the vicinity of 0.005), as previously seen in Fig 6E, which varies *σ* while holding the selection factor fixed at *s* = 1.

In summary, evolutionary simulations indicate that strong synergistic epistasis can underlie disease that arises in populations evolving on a generalized LP landscape. Neither disease nor epistasis tend to arise frequently, but when they do, they correlate; moreover, the strength of that correlation is a function of the severity of the disease. As a result, epistatic, rather than additive, effects tend to provide the most likely explanation for sufficiently severe disease. The level of severity (fitness loss) required for this is sufficiently small that causal alleles may even rise to relatively high frequencies (e.g. >1%).

As we found when exploring the dynamics of populations as a whole, the degree of phenotypic noise and the interaction parameter, *k*, have important effects on the degree to which epistasis and disease correlate. Interestingly, when *k* is small, it has a greater effect on the prevalence and magnitude of epistasis within a population (S6 Fig) than it does on localizing the population to the corner (S4 Fig). This effect arises because the local shape of the small portion of the landscape’s corner actually occupied by the population, which depends sensitively on *k*, matters more for the epistasis realized in individuals of the population than for the population dynamics as a whole. Put another way, moderate nonlinearities are sufficient to localize the population to the most nonlinear part of the landscape, but large nonlinearities are needed to generate strong epistasis in severely affected cases.

### Accounting for the Most Severe Common Diseases

We have seen how strong epistasis can arise in severely affected cases (those with low fitness), but how does the disease severity that arises in our simulations compare to that measured in common, heritable diseases? Averaging fitness (and epistasis) over all cases at each sampled time point reveals that diseased individuals in our simulations are most likely to have an average fitness of at least 0.8, relative to that of healthy controls (Fig 6H). For many clinically significant disorders, fitness is very likely in this range (e.g. bipolar disorder in females), but some common diseases of particular interest to human geneticists affect fitness much more severely, e.g. the average relative fitness of individuals with schizophrenia is 0.23 for men and 0.47 for women [27].

One candidate explanation for such severe, yet common, disease is rooted in the hypothesis that rare gene variants cause complex disease [28–30]. According to this hypothesis, there is a large, heterogeneous class of susceptibility variants at genes associated with disease, effectively elevating the overall mutation rate (e.g. above what we typically use in simulations), thus increasing the proportion of unfit individuals at mutation-selection balance. While S8 Fig confirms the predicted effect of increased mutation rate on the proportion of unfit individuals, it is accompanied by a sudden and drastic reduction of epistasis, presumably associated with a destabilization of the corner (cf. Fig 2D, which exhibits a similar corner destabilization when mutation step size is increased). Explaining the most severe common diseases by invoking a high effective mutation rate, it would seem, largely rules out epistasis as an important factor in the molecular underpinnings of those diseases. Accordingly, it is worth considering whether there are any other ways to explain the occurrence of such diseases at high prevalence in a population, that do not make the existence of strong epistasis unlikely.

One possibility relates to the potentially highly polygenic nature of some disease phenotypes, i.e. the degree to which genetic variants at a large number of different genes are collectively involved in causing disease. So far we have considered only an LP landscape defined by just two genes, in which only pair-wise epistasis can evolve. Yet, there is by now considerable evidence in favor of the existence of higher-order epistasis both in experimental systems [11,31–33] and humans [34]. Because the average fitness disadvantage associated with any individual allele would be expected to go down as the number of alleles with which it must interact goes up, one might expect that deleterious, high-order epistatic interactions would be less likely to be eliminated by natural selection than low-order ones, with the result that more severe epistatic disease could persist.

To test this idea, we evolved populations on an LP landscape defined by an arbitrary number of input values, each of which was determined by a different genetic locus. S9 and S10 Figs show that increasing the number of inputs (and genes) beyond the two considered thus far does not significantly alter relative case fitness.

Not only do evolutionary simulations subject to a larger number of causative genes show no increase in disease severity, they also demonstrate that synergistic epistasis between three or more genes doesn’t arise spontaneously, despite the fact that the phenotypic landscapes being considered allow for epistasis among as many as six genes at a time. This surprising result is most apparent as we vary levels of total phenotypic noise, as in S9 Fig. These data show that, while it is possible to identify conditions in which case-control phenotype differences are almost entirely due to epistasis, i.e. <*H*_*ind*_*-h*_*ind*_> > 0.8, and it is possible to localize the population to the corner with respect to all input values, it is not possible to achieve both simultaneously. Instead, the intermediate levels of phenotypic noise that favor epistasis (S9A Fig; see also Fig 6E) are insufficient to corner-localize the population with respect to more than 2–3 genes at a time (S9B Fig). In summary, at mutation-selection balance, increasing the number of inputs (i.e. the number of potential interacting genes) contributing to the LP model does not increase the contribution of epistasis to explaining disease phenotypes. However, if there were other forces keeping the population localized to a higher-dimensional corner even at modest noise levels, increasing the number of interacting genes could lead to an increase in disease epistasis.

We next turned to considering out-of-equilibrium scenarios. It has been suggested that severe disease often originate from a recent environmental shift, to which modern populations are still adapting [35]. Since our simulations already model environmental fluctuations as coordinated changes to input values, one way to simulate an abrupt environmental shift is to compute case relative fitness immediately following a large perturbation to the input values. S11 Fig shows the distribution of average case fitness (and epistasis) under such a scenario for a variety of shift magnitudes. As expected, these perturbations tend to reduce case fitness, but the effect saturates at large shift magnitudes because both cases *and* controls suffer fitness reductions. We reasoned that this saturation of disease severity would be circumvented if, instead of input value, we shifted selection strength, *s*, upward. The consequent narrowing of the ridge in the fitness landscape should leave optimal-fitness controls unscathed while dramatically reducing case fitness. As expected, a 5-fold increase in selection strength transiently increases disease severity (compare Fig 6I with 6H), attaining values comparable to those seen in schizophrenia at larger-fold selection shifts (S12 Fig). Importantly, performing the environmental shift at the level of fitness, instead of phenotype, implies that our prior conclusion—that epistasis is enriched in the most severely affected cases—remains intact (Fig 6I).

### Beyond LP Models

**Fig 7:**
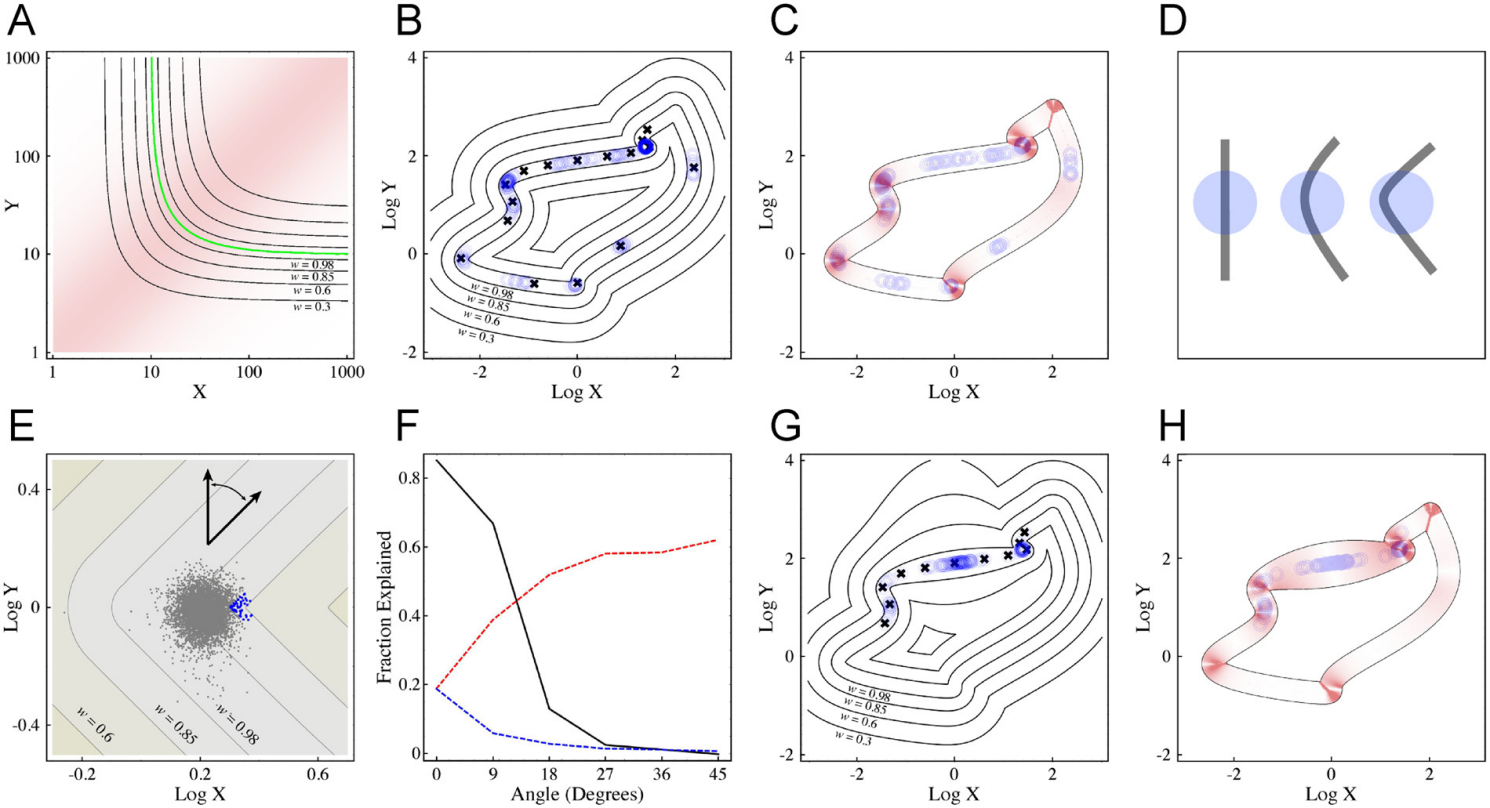
Evolutionary trajectories and epistasis on fitness ridges. (A) A variety of biologically realistic scenarios produce landscapes with continuous fitness ridges that resemble those in the LP model. Here we show a landscape for *Z* = *XY*/(*X*+*Y*), which models a scenario in which two gene products work together, but with a cost associated with expressing each. Compare with Fig 3A and 3B. (B) Evolution on an arbitrary fitness ridge-landscape, in which the optimal phenotype (*w* = 1) is defined by a parametric curve that traces a closed loop in the *X-Y* plane: ln(*X*) = sin(2*t*) cos(2*t*)– 2 cos(*t*), ln(*Y*) = (cosh(*t*) + 5 sin(*t*))/4, -*π* < *t* < *π*, and for points elsewhere in the plane, fitness falls off as a function of the shortest distance to the parametric curve. Black crosses indicate initial positions of populations chosen for 22 independent simulations. A combination of developmental and environmental noise was used (*σ*_*dev*_ = *σ*_*env*_ = 0.05, as in Fig 6C). Blue circles show, for each simulation run, the population mean values of ln(*X*) and ln(*Y*) at 11 timepoints, sampled once every 500,000 generations between 5,000,000 and 10,000,000 generations. The results show that corners in the fitness ridge function are attractors: populations that start at a corner tend to stay there, while those starting near enough to a corner are drawn there. At three locations along the curve, there are two corners in close proximity: (ln(*X*), ln(*Y*)) ≈ (-1.5, 1.25), (0, -0.75), and (1.5, 2). Nearby populations are always drawn more strongly to the sharper of the two corners. (C) Maximum epistasis in the vicinity of a high-fitness ridge. This figure shows only the region of the fitness landscape from Fig 7B where *w* > 0.95. Locations of blue circles are identical to Fig 7B, representing sampled population locations at late time points. The red shading indicates the maximum possible epistasis at any given point, derived from Eq. (S85) in S1 Text. Epistasis is maximized in the vicinity of corners in the fitness ridge, and minimized where the *X-Y* slope (orientation) of the ridge is close to vertical or horizontal. (D) Conceptual explanation for the attractiveness of corners. Because noise forces populations to sample from nearby regions of the fitness landscape, its effect may be viewed as a form of local averaging. As sufficiently thin fitness ridges may be conceptualized as segments of curves in the *X-Y* plane (black), the average value of fitness within any region (blue) may be equated with the arc-length of the optimal fitness contour that is contained within it. For simple regions, the greater the curvature of the contour, the more arc length can usually be contained within it. (E, F) Influence of ridge orientation on epistasis. In D, an LP-like fitness landscape (*s* = 1; *z*_*opt*_ = 1, as in Fig 6C) was rotated by 45°, so that its arms become diagonal, while the orientation of the corner (slope of the optimal fitness contour at the corner) becomes vertical (the angle of rotation is indicated by the arrows). A snapshot of an evolutionary simulation (*σ*_*dev*_ = *σ*_*env*_ = 0.05) is shown; blue and gray dots representing “case” and “non-case” individuals, respectively. As expected, the population localizes near the corner, but the individuals with the highest phenotype values (cases) are outliers only in X relative to the rest of the population. Panel E summarizes a variety of simulation runs in which the same fitness landscape was gradually rotated from its original orientation (0°) to 45°. The black curve gives the fraction of case-control phenotype differences attributable to epistasis (for cases with relative fitness < 0.9; i.e. the same quantity plotted in Fig 6E and 6G). The red and blue dashed lines indicate the mean fraction of the phenotypic variance of the population that is attributable to variance in *X* and *Y*, respectively. Note that epistatic explanation for disease vanishes as the corner orientation moves from diagonal to vertical. At the same time, phenotypic variance goes from being bi-genic to effectively monogenic. (G) Effect of variable fitness fall-off. In this simulation, the fitness landscape in B was distorted by spreading apart the fitness contours in a relatively straight part of the landscape lying between two corners. The meanings of black and blue symbols are as in panel B. The results show that locally flattening the fitness landscape destabilizes the corner at (-1.5, 1.25). However, the sharper corner near (1.5, 2) continues to be a strong attractor. The evolutionary simulations presented in this figure are described in greater detail in Methods. (H) Maximum epistasis in the vicinity of a high-fitness ridge. The figure presents a map of maximum possible epistasis as a function of location along the high fitness ridge in panel G, using the same approach as in panel C. Here we see that the locations to which populations are drawn by “survival of the flattest” may display little or no potential for epistasis.

The LP model was introduced as a mathematical representation of biochemical scenarios in which the quantity of interest is the concentration of a complex consisting of two (or more) gene products [23]. It is, however, mathematically equivalent to any scenario in which the lesser of two (or more) quantities determines function, as may easily occur when cellular components, networks, or even cells have to work together. Moreover, fitness landscapes similar to those produced by the LP model result from a variety of more general cases of gene interaction, particularly when there are tradeoffs between what genes accomplish collectively versus separately. For example, if we have two interacting gene products, *X* and *Y*, with fitness being increased by the amount of the product of [*X*] and [*Y*], but then also decreased separately by [*X*] and [*Y*] (for example, if there is some energetic cost associated with expressing both X and Y), then we easily produce landscapes such as that in Fig 7A (*Z = XY/*(*X+Y*)), which greatly resembles those in Fig 3A and 3B. Yet another way to produce a similar landscape is to have two interacting processes each of which is the outcome of a process that is a saturable function of a single gene (i.e. *Z* = (*X/*(*1+X*))*(*Y*/(*1+Y*)).

Such landscapes form continuous ridges in fitness-space because they represent scenarios in which fitness tradeoffs create a continuum of options for achieving a single objective. Because tradeoffs are thought to be such an important force in biological evolution, we think it particularly relevant to understand how populations evolve on ridge-landscapes. To approach this problem in the most general way, we began by finding criteria that could describe the local shapes of all possible ridge-landscapes in three dimensions (i.e. when fitness is a function of two genes, *X* and *Y*). We identified three such criteria, which we refer to as orientation, curvature, and fall-off. The first two are characteristics of the *X-Y* curves of constant fitness value, i.e. the fitness contours, with orientation meaning the slope (in the *X-Y* plane), and curvature being the inverse of the radius of curvature. Fall-off refers to how fitness changes as one moves orthogonal to a fitness contour (i.e. the spacing between contours). It may be characterized by the degree to which it is or is not locally uniform (spacing between contours relatively constant) or locally symmetric (fitness declining equally on both sides of the optimal fitness contour).

We began by investigating arbitrary landscapes in which fall-off is symmetric and relatively uniform along the entire optimal fitness contour (the LP model falls into this category), and later explored the effects of varying fall-off. Populations were initiated at various locations, and evolution simulated in the same way as was done for the LP model (see Methods). Parameters that were varied included the strength of developmental and environmental noise, the strength of selection, and the mutational step size.

Fig 7B shows the results on one such landscape, in which the fitness contours are displayed. Black crosses show the *X*-*Y* values at which populations were initiated, while blue circles show population mean locations 5,000,000 generations later, and for a series of 500,000-generation time points thereafter. From a large number of analyses such as these we noticed two consistent trends.

First, in the presence of noise, evolutionary stability is closely related to the curvature of the optimal fitness contour, with populations typically being drawn to locations of highest curvature. These locations correlate strongly with locations at which the potential for epistasis is greatest (Fig 7C). This is the same behavior we observed in the LP model, in which noise drives populations to the corner, and, generally speaking, the sharper the corner (which corresponds to lower values of the parameter *k* in the LP model), the stronger the effect. We may rationalize this behavior by the fact that phenotypic noise enforces a form of spatial averaging over a region of the fitness landscape (as described above and in S1 Text, section 3). On an arbitrary fitness ridge of uniform width, “effective fitness” will depend on the area under the ridge that is contained within a given averaging radius, which will naturally be a function of the ridge’s curvature. In general, the greater the curvature, the more arc-length of the optimal fitness contour will be contained within the averaged area (Fig 7D). Thus, the notion that evolution drives populations to curves—i.e. “survival of the curviest”—can be expected to apply broadly, not just to LP models or landscapes that resemble them.

Second, we also found that, as in the LP model, the emergence of epistasis as a major factor in determining the phenotypes of low-fitness individuals correlated with the arrival of populations at regions of high curvature. But, this correlation was no longer perfect. At some corners, even sharp ones, little epistasis was observed. A deciding factor, we realized, was the orientation (slope) of the optimal fitness contour (at the point of highest curvature). When populations were at locations where orientation was approximately diagonal (as it always is at the corner of LP landscapes), a large proportion of the phenotype of the most unfit individuals could be explained by epistasis. When orientation was close to horizontal or vertical, almost none was. We can rationalize this behavior by noticing that the slope of the optimum fitness contour is a measure of the relative contributions of changes in *X* and *Y* to phenotype. When that slope parallels one of the two axes, phenotype is necessarily insensitive to small changes along that axis. Epistasis between *X* and *Y* becomes negligible because phenotypic variation correlates significantly with genetic variation at only one of the two loci. We can illustrate this graphically (Fig 7E and 7F) by rotating the LP landscape about the origin to various extents, evolving populations on them, and observing that, as the fraction of the phenotypic variance explained, individually, by *X* and *Y* goes up toward one, the fraction of the case-control phenotype differences attributable to epistasis, for cases with relative fitness less than 0.9 (the same quantity plotted in Fig 6E and 6G), goes down toward zero. As described in S1 Text, we can more generally analyze the effects of orientation and curvature on epistasis by deriving general expressions for orientation, curvature, and maximum possible epistasis in terms of the partial derivatives of the phenotype with respect to *X* and *Y*. Such analysis reveals the generality of the relationship between orientation and potential for epistasis, and specifies the exact conditions under which curvature and epistasis will correlate with one another.

After verifying these findings over a variety of arbitrary ridge shapes, we considered the effects of varying fall-off. As shown in Fig 7G, by appropriately adjusting the spacing between fitness contour lines, it is possible to find scenarios in which evolution in the presence of noise drives populations away from regions of highest curvature, and toward regions of low potential for epistasis (Fig 7H). Such simulations reveal that evolutionary dynamics in the presence of noise reflects two “forces” that can be at odds with each other: survival of the curviest, as described above, and “survival of the flattest”. The latter has been described previously, as a means of explaining how evolution selects for robust, or canalized, outcomes [36]. Like survival of the curviest, survival of the flattest arises from the fact that, in the presence of noise, “effective fitness” reflects a local averaging of the fitness landscape. A population may increase that average fitness either by moving to a landscape location that is curvier (Fig 7D) or flatter (Fig 7G), depending upon which feature exerts the stronger attraction, but only in the former case may such movement be expected to produce a strong increase in epistasis. Thus, even though the conclusions drawn here from the analysis of LP landscapes may apply to a broad range of biological scenarios, we should not expect them to apply to all (see Discussion).

## Discussion

Human populations are ill-suited for the direct, empirical discovery of epistasis [6], which is why theory and simulation currently play a major role in the debate about whether epistasis is an important factor in the genetic etiology of human variation, e.g. [37,38]. Here we used a combination of mathematical analysis and evolutionary simulation to investigate the conditions under which the evolutionary forces of drift and natural selection tend to cause relatively common, deleterious traits (“common disease”) to be due substantially to epistasis. A central aspect of our analysis was the inclusion of phenotypic “noise”, i.e. randomness in the genotype-phenotype map either at the level of individuals (developmental noise) or at the level of the map itself (environmental noise).

It has long been appreciated that noise can favor the evolution of canalization [39,40], although the relationship between, and relative importance of, different kinds of noise continue to be debated [41–43]. Moreover, noise-driven canalization has previously been observed to correlate with an increase in epistasis. For example, when evolving digital organisms *in silico*, Wilke and Adami noted that at sufficiently high mutation rates—effectively a form of long-term phenotypic noise—populations tended to be drawn to flatter regions of the phenotypic landscape (“survival of the flattest”), even if such regions were suboptimally fit, and this was accompanied by an increase in the prevalence of synergistic epistasis [44]. Qualitatively similar findings of mutational robustness emerging together with epistasis have come from simulated evolution of gene regulatory networks in the presence of recombination [15], and have been confirmed in experimental studies of the function of single enzymes in bacteria [45].

Because noise in the genotype-phenotype relationship may be viewed as a form of local averaging of the fitness landscape (see S1 Text), it is not surprising that several different kinds of noise—including developmental and environmental variability of the kind we consider here—have all been observed to drive evolving populations to flatter locations [46–48], where robustness to variation of any sort (genetic or environmental) will be buffered [47,49]. What has been unclear is whether such movement is necessarily accompanied by an increase in epistasis, and if it is, whether an identifiable relationship exists between the parameters of noise, the phenotypic landscape, and distribution of epistatic effects that arise among individuals.

In part to address these questions, we studied a class of simple landscapes defined by the generalized LP model, which may be viewed as an abstraction of several biologically relevant, mathematically related, gene-gene interaction scenarios. Rather than consider only average epistatic effects, we focused on the necessary conditions for especially unfit (“disease”) phenotypes to be explained by epistasis. These conditions included intermediate levels of phenotypic noise (Fig 6E), selection strength (Fig 6G), and mutation rate (S8 Fig), as well as a sufficiently nonlinear genotype-phenotype mapping (i.e. small *k*; S4 Fig). The extent to which selection drove populations to highly epistatic regions of the phenotypic landscape depended on the magnitude of the non-linearity (e.g., parameter *k* in S4 Fig), as well as the scale over which the population was spread—which depended on both the strength of selection, *s*, and phenotypic noise level, *σ* (through the product *sσ*^2^; Fig 6G).

When these conditions were met, synergistic interactions among both rare and common alleles (S5 Fig) tended to be a major cause of disease, particularly in the most severely affected individuals (Fig 6C), regardless of the number of genes underlying the trait in question (S10 Fig). That the most severely affected individuals are expected to be the most “epistatic” can be understood by noting that such individuals are farthest away, on the phenotypic landscape, from the norm, and therefore most influenced by the landscape’s nonlinearity. In agreement with this prediction, we note that, in recent yeast work that has identified substantial amounts of epistasis among standing genetic variants, analysis had been focused specifically on the most extreme phenotypes [11]. Although the individuals that possess such phenotypes may be rare, they can be better positioned to provide mechanistic insights into the phenotypic landscape on which an entire population resides.

When we subsequently extended our analysis to other kinds of two-gene fitness surfaces, all of which, like the LP model, had optimal-fitness ridges, we again observed evolving populations drawn by noise to the most sharply curved parts of ridges, a phenomenon we term “survival of the curviest”. Arrival at highly curved regions tended to correlate with an increase in robustness, as well as reduced phenotypic variance despite increased accumulation of genetic variation. Furthermore, as in the LP model, arrival at such locations also tended to correlate with an increase in observed epistasis. This was not always the case, however, as this pattern was violated in two types of situations:

First, when ridge corners (vertices) were oriented close to horizontal or vertical, populations were still drawn to them, but strong epistasis was not observed, as phenotypic variance essentially became a function of only one of the two genes (Fig 7E and 7F). In practical terms, localization of a population to this type of landscape feature ought to make the discovery of causal gene variants by traditional statistical means (e.g. GWAS) much easier, because it both reduces the number of genes at which variation has any effect, and eliminates all but “main effects”. Accordingly, to the extent that one cares primarily about heritable diseases that GWAS has failed to adequately explain, one should be biased against encountering this type of landscape feature.

Second, when we varied the way fitness declined along ridges, we found that “survival of the curviest” could sometimes be thwarted by”survival of the flattest”, with populations being drawn to locations at which two genes still contribute to phenotypic variance, but little epistasis occurs (Fig 7G and 7H). Such simulations show that the connection that has previously been noticed between selection for robustness and epistasis (e.g. [15,44]) is not, in fact, a necessary one (see also S1 Text, section 6, for an analytical treatment), and that our ability to generalize conclusions drawn from the study of LP landscapes to other biological settings depends upon whether landscapes in which flatness outweighs curviness are found frequently or rarely in biology. We note, in this regard, that the landscapes explored in Fig 7 were arbitrary curves, not meant to be representative of any specific biological processes. How easily networks of actual biological components achieve such topographies remains to be investigated.

It should be noted that none of the conclusions of the present study are necessarily in conflict with earlier work on the evolution of epistasis [15–18], including studies that have suggested that epistasis is unlikely to play a significant role in complex human traits in general, e.g. [14]. The reason is that we have focused here not on traits in general, but on a very particular subset, namely those that are substantially deleterious (e.g. fitness <0.9) and moderately (but not very) common (population frequency between 0.1 and 10%). We justify this focus by the fact that such criteria would appear to capture a great deal of clinically relevant human disease.

There are two reasons why this focus is so important. First, the situations in which both criteria (substantially deleterious and moderately common) are met are evolutionarily rare events (because natural selection disfavors the appearance of common disease). Accordingly, arguments based on the typical evolutionary behaviors of traits do not necessarily apply. In effect, we are not asking here about the probability of observing epistasis in general, but on its probability given prior knowledge of common disease.

Second, by restricting our view to disease frequencies of 10% or lower, our analysis operates in a regime in which functional epistasis has the potential to exist without much statistical epistasis (Fig 1). This means that the effects of epistasis on the evolutionary process itself can be very small (deflating the argument that epistasis *per se* is deleterious, and thus should be selected against, e.g. [37]). It also means that the impact of epistasis on patterns of heritability can be very small (which could likewise eliminate most arguments against the existence of epistasis based on the analysis of data, e.g. GWAS). The condition under which functional epistasis can be large, while statistical epistasis is negligible, is when populations are distributed on a phenotypic landscape that is relatively linear where the majority, “control” individuals are found, becoming non-linear where the minority, “cases” reside (see Fig 1). On the simple LP landscape, such a scenario occurs when populations are centered near the “corner”, i.e. when inputs from different loci are reasonably balanced. As we show here, the force that drives populations to that location is noise.

In the realm of human genetics, the use of unbiased genetic association studies to uncover explanations for complex human disease has often proved exceptionally challenging, leaving clinicians and researchers to decide how best to allocate further efforts. Prominent competing hypotheses have attributed undiscovered heritable variation variously to epistatic interactions among genes; to large numbers of variants of very small effect; or to variants currently excluded from the association studies. The latter include both rare variants and structural alterations other than SNPs, such as tandem-repeat polymorphisms [50], copy-number variants [51], and large translocations [52]. While such structural variants have been associated with individual diseases (e.g., [53]), they have yet to account for a great deal of the “missing” heritability of common diseases.

Here, by examining the conditions that favor the evolution of disease epistasis, we hoped to illuminate circumstances under which epistatic explanations are most likely. We believe that those circumstances, while not universal, are likely common enough to warrant additional effort toward discovering epistatic interactions. In this regard, we can imagine at least three specific ways in which the present study might be useful. First, it may help investigators use information about the social, cultural or historical aspects of a particular disease state to develop intuitions about the probability of an epistatic explanation. For example, expected interactions between mental illness and culture, or metabolic illness and diet, suggest that diseases in these categories are more likely to be evolving in the face of recently fluctuating environments than, say, congenital heart defects. Second, it could encourage investigators to directly measure developmental noise genome-wide, e.g., as reflected in variations in RNA-seq levels in tissues from individuals with the same SNP genotype [54], with the goal of identifying cases in which the observed noise is of the right magnitude to favor the emergence of epistatic, disease-causing interactions. Third, our simulations of the LP model suggest that risk alleles that combine to produce exceedingly unfit individuals (fitness < 0.75) ought to have frequencies that lie in the 0.2–0.5% range, at the low end of what is typically considered in GWAS studies, but higher than expected for rare alleles with Mendelian effects. Potentially, GWAS that focused exclusively in this allele frequency range might actually have greater power to detect epistasis than GWAS that considers all variants.

## Methods

Numerical calculations of linear regression were implemented in python and visualized in Matlab. Evolutionary simulations were implemented in C, as described below.

We simulated populations of *N* = 5000 diploid individuals forward in time using non-overlapping generations. An individual’s genotype consisted of two allele values at each locus. Each input value (e.g., *x* or *y*) was determined by a single locus, and was taken as the sum of the individual’s two allele values at that locus. We restricted allele values to be less than 100 (i.e. each input value was constrained to be less than 200). Every generation, then, each individual’s allele values were summed to determine its nominal input values. These values were adjusted first by environmental noise, which exerts the same effect on each individual in the population. The resulting values were then adjusted by developmental noise, applied independently to each individual.

The following Metropolis-Hastings algorithm was used to model persistence of environmental noise from one generation to the next, i.e. the temporal autocorrelation of the noise. Given that the environmental perturbation term in generation *i* was Δ*x*_*i*_, the perturbation in the next generation, Δ*x*_*i+1*_, was determined as follows. A proposed value of the perturbation, Δ*x*_*prop*_, was generated by adding a Gaussian-distributed random variate with mean zero and variance *σ*_*step*_^*2*^ to Δ*x*_*i*_. If Pr(Δ*x*_*prop*_) = *exp*[-Δ*x*_*prop*_^*2*^/(2*σ*_*env*_^*2*^)] was greater than Pr(Δ*x*_*i*_) = *exp*[-Δ*x*_*i*_^*2*^/(2*σ*_*env*_^*2*^)], then the proposed perturbation was accepted and Δ*x*_*i+1*_ was assigned the value Δ*x*_*prop*_. Otherwise, the proposed perturbation was accepted with probability proportional to the ratio Pr(Δ*x*_*prop*_)/Pr(Δ*x*_*i*_). If the proposed perturbation was rejected, then the environmental perturbation remained unchanged (Δ*x*_*i+1*_ was assigned the value Δ*x*_*i*_). This algorithm ensures that the steady-state probability that the environmental perturbation has a particular value, Δ*x*, is proportional to *exp*[-Δ*x*^*2*^/(2 *σ*_*env*_^*2*^)]. For given values of *σ*_*step*_ and *σ*_*env*_, the autocorrelation time was determined *a posteriori* from the autocorrelation function of Δ*x*_*i*_. For most of the results presented here, *σ*_*step*_/*σ*_*env*_ = 0.25, generating an autocorrelation time of ≈ 27 generations.

Having applied the phenotypic noise to the nominal input values, the resulting set of effective input values (*x*_*e*_, *y*_*e*_) was used to calculate the individual’s phenotype, *z*, and fitness, *w*. Simulations with *d* > 2 genes were performed in an analogous way, with the effective set of input values (*x*_*1e*_, *x*_*2e*_,… *x*_*de*_) determined by the application of environmental and developmental noise to each of the *d* nominal input values (*x*_*1*_, *x*_*2*_,… *x*_*d*_).

The next generation was then constructed by a method of sampling with replacement, where the probability of an individual being sampled to become a parent was proportional to its fitness. For each new offspring, two parents were identified using the following sampling-rejection scheme. First, a prospective parent was drawn at random from the entire population. Then, a uniform random number was drawn between zero and the highest fitness in the population. If the prospective parent’s fitness was higher than the random number, the parent was accepted. If not, they were rejected and a new random prospective parent was drawn. Sampling with replacement implies that a rejected parent could be redrawn and accepted later, and a parent could be drawn more than once; the only exception was that the same candidate could not be drawn for both parents of the same individual.

The offspring genotype was constructed by randomly selecting one of the two alleles at the first locus from each parent. A recombination parameter determined the probability of switching chromosomes between loci. In most simulation results presented in the text, we assumed free recombination, so that the two alleles at each locus were sampled with equal probability and with no correlation among loci.

Finally, a mutation was applied independently to each allele with probability *μ* = 10^−4^ per allele per generation. In the event of a mutation, a normally distributed quantity (mean zero, variance *σ*_μ_^2^) was added to the natural logarithm of the allele’s value (thereby ensuring that each allele value remains positive). If the mutation led to an allele value in excess of 100, the mutation was rejected, and the inherited allele retained its original value.

Each simulation was run for 10 million generations, and the first 1 million generations discarded. Populations were typically analyzed at intervals of 10,000 generations.

At the start of each simulation on the generalized LP landscape, each individual was homozygous, with allele values chosen so that *x* = *y* and *z* = 1, i.e. all individuals were positioned at the corner of the ridge in the phenotypic-noise-free fitness landscape. For example, when *k* = 0, the initial value associated with each allele was 0.5, so that *x* = *y* = 1.

For evolutionary simulations on arbitrary, non-LP landscapes (Fig 7), a lookup table of phenotype values was pre-computed based on a grid of combinations of log-transformed underlying gene values (ln(*x*) and ln(*y*)). During the course of simulation, individual phenotype values were calculated by linear interpolation from the four closest ln(*x*), ln(*y*) combinations from the lookup table. Fitness values were then calculated according to (Eq 2) in the main text.

For Fig 7B and 7C, the phenotype lookup table was constructed as follows. An array of pairs, (*ln*(*x*), *ln*(*y*)) was constructed consisting of 2100 values of ln(*x*) from -3.5 to 3.5 by 1800 values of *ln*(*y*) from -2 to 4. For each pair, the logarithm of the phenotype value *z* was taken to be a multiple of the distance *d* to the closest point on the parametric curve corresponding to the optimal phenotype, *z*_*opt*_ = 1. For points outside the closed curve, *ln*(*z*) = –*d*, so that *z* < *z*_*opt*_; for points inside the closed curve, ln(*z*) = *d* so that *z* > *z*_*opt*_.

For Fig 7G and 7H, the phenotype lookup table was constructed as above, but with the following modification. When calculating the distance *d*, we introduce a scaling factor *f* that depends on the curve parameter *t* (defined by the point on the curve nearest to (ln(*x*), ln(*y*))). For *t* <*π*/3 or *t*>2*π*/3, *f* is set to 1. But for *π*/3 < *t* < 2*π*/3, *f* is set to (3 –cos(6*t*))/2. This reduces the calculated distances in the vicinity of *t* = *π*/2, meaning that phenotype and fitness values change less rapidly in response to changes in *x* and *y*.

## Supporting Information

S1 FigMeasures of epistasis.(A, B) Low statistical epistasis can exist in the presence of high functional epistasis. Statistical epistasis was computed using either all of the genotypes in a population (red), or a biased version of that distribution in which the prevalence of individuals with large phenotype values was deliberately increased (green). In this example, the prevalence of individuals displaying the top 1% of phenotypes was increased to 50%, as would typify a balanced case-control study of a common disease. Eq (S34) defines the mathematical relationship between the distributions of genotypes in the original and adjusted populations. Panels A and B differ in the functional form of the phenotypic landscape, corresponding to different values of the parameter *n* in the definition, $Z(x,y)=x+y+\frac{{\left(xy\right)}^{n}}{\xi^{2n-1}}$; the lines in each panel were generated by varying the parameter ξ, which controls the allele values at which the landscape crosses over from relatively linear to highly nonlinear. The population distribution was defined by $p(x,y)=\frac{1}{\lambda^{2}}e^{-(x+y)/\lambda }$ with *λ* = 0.2. Functional epistasis was averaged over all case individuals. The results show that enriching for case genotypes, which have the greatest levels of functional epistasis, does not necessarily increase statistical epistasis, and often reduces it. (C) Though functional epistasis of cases is not sufficient to generate high levels of statistical epistasis (panels A and B), the latter can result from moderate levels of functional epistasis in the bulk of the population, as measured by evaluating functional epistasis at one standard deviation of the (original) genotype distribution, 1 –*h*_ind*_(λ,λ | 0,0). The data also show that statistical epistasis depends on the spread of the population, *λ*, and the shape of the phenotype landscape, ξ, only through the lumped parameter, 1 –*h*_ind*_(λ,λ | 0,0) (derived in Section 1.2 in SI text). Line is defined by Eqs (S27)–(S29); circles are numerical estimates for a set of (ξ, *λ*) values. *n = 2*.(PDF)

S2 Fig Distribution of nominal and effective input values under developmental and environmental noise and under case-control sampling. (A-D) Distribution of input values among all individuals (green) in a simulated population and conditioned upon individuals being cases (pink) or controls (dark blue). Controls are drawn from the middle 50% of rank-ordered phenotype value (ranging from the 25^th^ to the 75^th^ percentile); cases represent the top 1% of this rank-ordered distribution. Since developmental noise is uncorrelated among individuals, the *nominal* input values of cases and controls are poorly differentiated (A), implying little heritability. Environmental noise, on the other hand, is correlated across individuals, resulting in a correlation between *nominal* input values and case-control status (C), and high heritability. (E-H) Time courses. Blue lines connect points corresponding to the average values of *x* and *y* in the population at intervals of 500,000 generations. Red bars indicate the magnitude of the variation in the population at each sampled timepoint (+/-2 standard deviations). (I-L) Median locations of case (pink) and control (dark blue) samples for the same temporal sequence of samples shown in panels E through H. Black lines connect control and case medians from the same time point. Parameter values are *z*_*opt*_ = *s* = 1, *N* = 5000, *k* = 0.01, and *σ*_μ_ = *σ*_*dev*_ = *σ*_*env*_ = 0.1
(PDF)

S3 Fig Random walk on the LP landscape. (A) Evolutionary path of the population center of mass as a function of input values. Selection is governed by the LP fitness landscape, with hotter colors (white, yellow) and colder colors (red, black) representing high and low fitness values, respectively. When interaction strength is weak (*k*>>1), as shown here, the corner is evolutionarily unstable. (B) When the interaction strength is strong (*k*<<1), the population follows an unbiased random walk along the arms, except in close proximity to the corner, where it tends to localize. (C) The random walk of the population center of mass along the horizontal arm is biased towards the corner by the graded effective fitness profile, *w*_*e*_(*x*), where *x* represents the natural logarithm of the input value. (D) *g*(*x*|*x*′) is the probability density that a mutation changes (the natural log of) the value of the gene to *x*, given that (the natural log of) the input value prior to the mutation was *x*′. (E) The probability, ρ(*x*|*x*′), that a mutant allele with value *x* fixes in a population of alleles of value *x*′ (again in log space), corresponding to the effective fitness profile shown in (C). In C–E, the horizontal axis represents the *natural logarithms* of the corresponding input values. *N* is population size.
(PDF)

S4 Fig Phenotypic noise stabilizes the corner at smaller *k*. Fraction of simulations in which the population-mean ratio of the two input values lay within the indicated bounds (1.2 for A and B, 1.4 for C and D, and 3.0 for E and F). Parameter values were *z*_*opt*_ = *s* = 1 and *N* = 5000. At moderate noise levels (A, C, and E), environmental and developmental noise behave similarly in terms of localizing the population at the corner. At higher noise levels (B, D, and F), developmental noise localizes the population to the corner more effectively than does environmental noise.
(PDF)

S5 Fig Population frequencies of alleles causing disease. Each case individual is represented as a single point whose color indicates the population frequency of the rarest allele carried by that individual. (Recall that the phenotype value *z* depends on the values of an individual’s two alleles at each of two loci.) Results are pooled over the five timepoints in one simulation run with the lowest mean fitness of cases relative to controls. Parameter values are σ_*env*_ = σ_*dev*_ = 0.05, *s* = 1, and *k* = 0.
(PDF)

S6 Fig How epistasis depends on the interaction constant, *k*. Heat maps with intensity representing the epistatic fraction, *H*_*ind*_*—h*_*ind*_, averaged over all cases with relative fitness < 0.9 and all sampled time points for *k* = 0 (A) and *k* = 0.01 (B). The combinations of environmental and developmental noise levels that lead to significant epistasis are similar in the two cases, but stronger interaction (i.e. lower value of *k*) results in a greater fraction of the case-control phenotype difference being attributable to epistasis.
(PDF)

S7 Fig Low relative fitness predicts epistasis among cases. Summary of the data in Fig 6C. Circles indicate the median value of epistasis fraction, *H*_*ind*_*—h*_*ind*_, for case individuals with similar fitness values, and error bars indicate the corresponding 25^th^ and 75^th^ percentile values.
(PDF)

S8 Fig How mutation rate, *μ*, affects epistasis and case fitness. Mutation rates (per allele per generation) for each row are indicated on the right-hand side. The left-hand panels show the joint distribution of the epistatic fraction, *H*_*ind*_*—h*_*ind*_, and fitness (relative to mean control fitness) over all cases and all sampled time points. In the right-hand panels, each dot represents a single time point at which the epistatic fraction and relative fitness were averaged over all cases. Parameter values are *σ*_*dev*_ = *σ*_*env*_ = 0.05 and *σ*_μ_ = 1.0.
(PDF)

S9 FigEffect of dimensionality (number of inputs) on disease epistasis and corner localization.(A) Epistatic fraction *H*_*ind*_−*h*_*ind*_ averaged over all cases with a relative fitness of less than 0.9 and over all time points, as a function of the total phenotypic noise level $\sigma =\sqrt{\sigma_{dev}^{2}+\text{​}\sigma_{env}^{2}}$, illustrating the biphasic dependence of epistasis on noise level evident in Fig 6E and 6G. Epistasis decreases more with increasing developmental, versus environmental, noise (compare second and third rows). Increasing the dimensionality of the system has only a modest effect on the epistatic fraction, with the largest effect occurring when *σ*_*env*_ > *σ*_*dev*_. (B) The number of inputs that are localized to the corner, defined as the number of time- and population-averaged input values that are within one mutational step (*σ*_μ_) of the corner, as a function of phenotypic noise level, $\sigma =\sqrt{\sigma_{dev}^{2}+\text{​}\sigma_{env}^{2}}$. Greater values of phenotypic noise are required to localize larger numbers of dimensions to the corner. As seen in Fig 5, increasing developmental noise monotonically enhances corner localization (second row) while intermediate levels of environmental noise correspond to maximum corner localization (third row). In both columns, rows differ in the relative contributions of *σ*_*dev*_ and *σ*_*env*_ to *σ*.(PDF)

S10 Fig Effect of dimensionality (number of inputs) on epistasis and case fitness. Each row corresponds to a different number of inputs in the LP model (indicated on the right-hand side). Each input value is determined by its own locus, and all loci are unlinked. The first and third columns show the joint distribution of the epistatic fraction, *H*_*ind*_*—h*_*ind*_, and fitness (relative to mean control fitness) for all cases and all sampled time points. In the second and fourth columns, each dot represents a single time point, at which the mean values of the epistatic fraction *H*_*ind*_−*h*_*ind*_ and case relative fitness were calculated. The first and second column (*σ*_*dev*_ = *σ*_*env*_ = 0.05) correspond to the condition under which we observed the strongest epistasis among cases with relative fitness < 0.9 (the peak in the first row of S9A Fig). The third and fourth column (*σ*_*dev*_ = 0.05; *σ*_*env*_ = 0.15) correspond to the condition under which dimensionality appeared to have the greatest influence on the epistatic fraction for cases with relative fitness < 0.9 (*σ* = 0.158 in third row of S9A Fig).
(PDF)

S11 Fig Effect of sudden environmental shift on case epistasis and fitness. Simulations were run as described, but in this case an additional environmental shift was temporarily performed immediately prior to sampling case and control individuals at each time point (represented by points on the graphs). Environmental shifts were implemented by increasing both input values by a fixed quantity Δ_*env*_ on a logarithmic scale (*e*.*g*., ln*x* became ln*x* + Δ_*env*_). Shifting the environment in this way reduces the fitness of cases relative to controls, but the reduction is limited by the fact that both cases and controls suffer a fitness cost at large environmental shifts. Parameter values were *k* = 0; *s* = 1; *σ*_*dev*_ = *σ*_*env*_ = 0.05; *σ*_μ_ = 1; *μ* = 10^−4^.
(PDF)

S12 Fig Effect of a sudden increase in selection on case epistasis and fitness. Simulations were run with selection strength *s* = 1 (top row). At each time point, we temporarily increased the strength of selection from *s* to *s’* before averaging epistasis, *H*_*ind*_*—h*_*ind*_, and relative fitness over all cases (second through third rows). Under pure developmental noise (left column, top row), all cases have reduced fitness relative to controls, reflecting the fact that the population is centered at the corner. In contrast, under pure environmental noise (right column, top row), values of case fitness extend upwards to 1 (and beyond), reflecting the fact that the population is not necessarily centered on the corner. That is, when environmental noise shifts the population so that most of its individuals have sub-optimal phenotype values, cases, which represent the individuals with the highest phenotype values, will have higher fitness than controls. Since increasing the strength of selection has little effect on high-fitness individuals, it can have a substantial impact on the relative fitness of cases and controls (second through third rows). Parameter values were *k* = 0 and *σ*_μ_ = 1.
(PDF)

S1 Text Formulation and analysis of mathematical models. (PDF)
