# Supplementary figures and images for: Survival of the Curviest: Noise-Driven Selection for Synergistic Epistasis
Source: PLoS Genet. 2016 Apr 28;12(4):e1006003. doi: 10.1371/journal.pgen.1006003 (PMC4849581; doi:10.1371/journal.pgen.1006003)

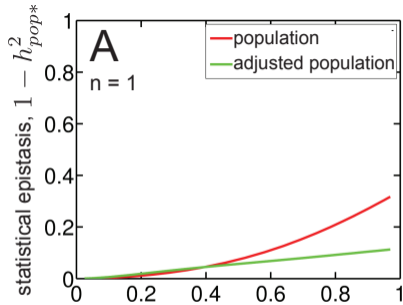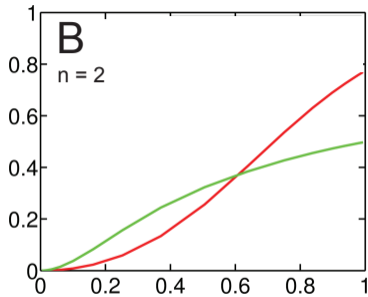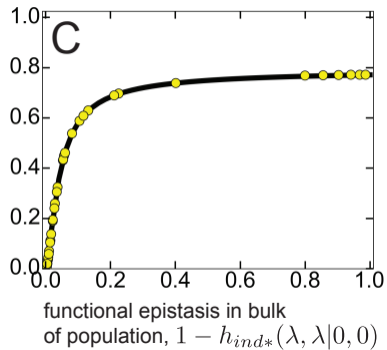

Supplement: S1 Fig — (A, B) Low statistical epistasis can exist in the presence of high functional epistasis. Statistical epistasis was computed using either all of the genotypes in a population (red), or a biased version of that distribution in which the prevalence of individuals with large phenotype values was deliberately increased (green). In this example, the prevalence of individuals displaying the top 1% of phenotypes was increased to 50%, as would typify a balanced case-control study of a common disease. Eq (S34) defines the mathematical relationship between the distributions of genotypes in the original and adjusted populations. Panels A and B differ in the functional form of the phenotypic landscape, corresponding to different values of the parameter n in the definition, Z(x,y)=x+y+(xy)nξ2n−1; the lines in each panel were generated by varying the parameter ξ, which controls the allele values at which the landscape crosses over from relatively linear to highly nonlinear. The population distribution was defined by p(x,y)=1λ2e−(x+y)/λ with λ = 0.2. Functional epistasis was averaged over all case individuals. The results show that enriching for case genotypes, which have the greatest levels of functional epistasis, does not necessarily increase statistical epistasis, and often reduces it. (C) Though functional epistasis of cases is not sufficient to generate high levels of statistical epistasis (panels A and B), the latter can result from moderate levels of functional epistasis in the bulk of the population, as measured by evaluating functional epistasis at one standard deviation of the (original) genotype distribution, 1 –hind*(λ,λ | 0,0). The data also show that statistical epistasis depends on the spread of the population, λ, and the shape of the phenotype landscape, ξ, only through the lumped parameter, 1 –hind*(λ,λ | 0,0) (derived in Section 1.2 in SI text). Line is defined by Eqs (S27)–(S29); circles are numerical estimates for a set of (ξ, λ) values. n = 2. (PDF) [file pgen.1006003.s001.pdf]

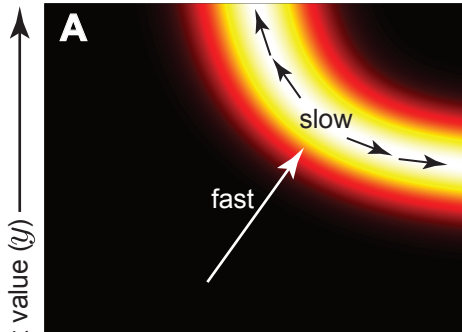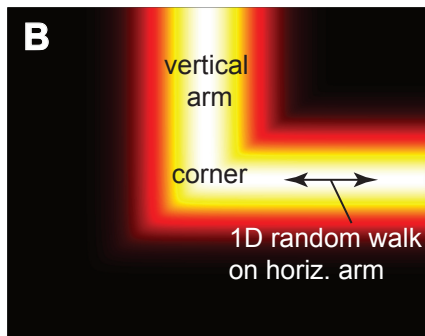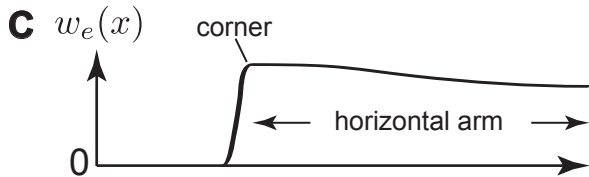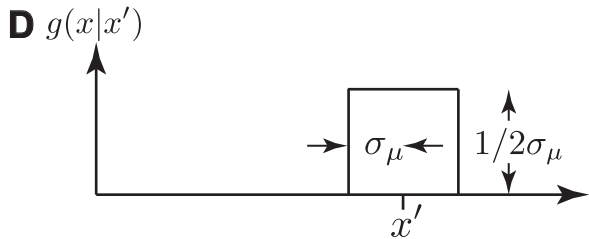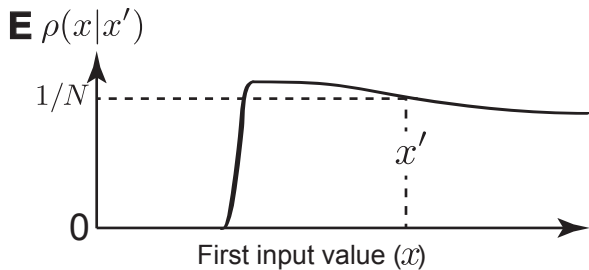

Supplement: S3 Fig — (A) Evolutionary path of the population center of mass as a function of input values. Selection is governed by the LP fitness landscape, with hotter colors (white, yellow) and colder colors (red, black) representing high and low fitness values, respectively. When interaction strength is weak (k>>1), as shown here, the corner is evolutionarily unstable. (B) When the interaction strength is strong (k<<1), the population follows an unbiased random walk along the arms, except in close proximity to the corner, where it tends to localize. (C) The random walk of the population center of mass along the horizontal arm is biased towards the corner by the graded effective fitness profile, we(x), where x represents the natural logarithm of the input value. (D) g(x|x′) is the probability density that a mutation changes (the natural log of) the value of the gene to x, given that (the natural log of) the input value prior to the mutation was x′. (E) The probability, ρ(x|x′), that a mutant allele with value x fixes in a population of alleles of value x′ (again in log space), corresponding to the effective fitness profile shown in (C). In C–E, the horizontal axis represents the natural logarithms of the corresponding input values. N is population size. (PDF) [file pgen.1006003.s003.pdf]

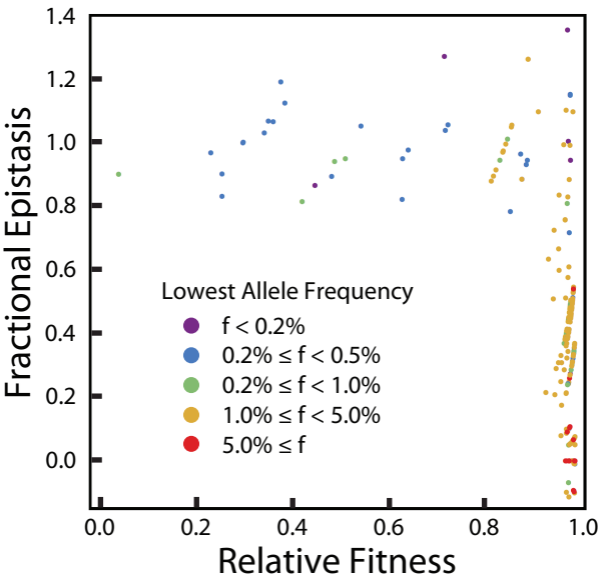

Supplement: S5 Fig — Each case individual is represented as a single point whose color indicates the population frequency of the rarest allele carried by that individual. (Recall that the phenotype value z depends on the values of an individual’s two alleles at each of two loci.) Results are pooled over the five timepoints in one simulation run with the lowest mean fitness of cases relative to controls. Parameter values are σenv = σdev = 0.05, s = 1, and k = 0. (PDF) [file pgen.1006003.s005.pdf]

Mean Epistatic Fraction,  $\langle H_{ind} - h_{ind} \rangle$

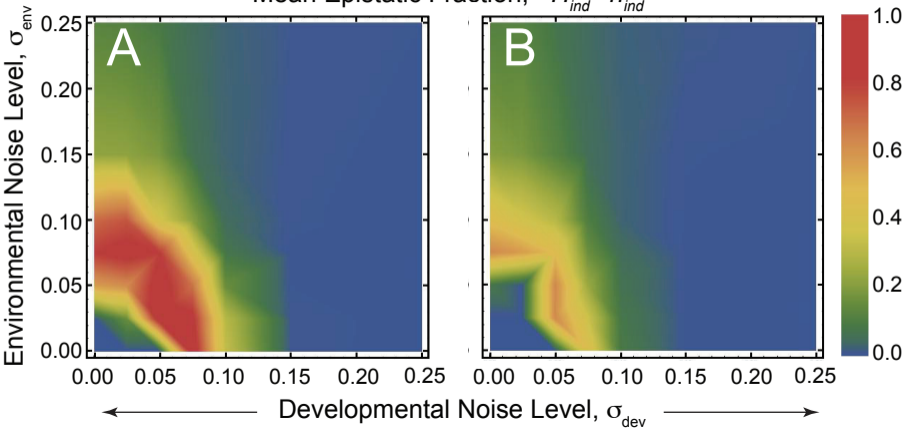

Supplement: S6 Fig — Heat maps with intensity representing the epistatic fraction, Hind—hind, averaged over all cases with relative fitness < 0.9 and all sampled time points for k = 0 (A) and k = 0.01 (B). The combinations of environmental and developmental noise levels that lead to significant epistasis are similar in the two cases, but stronger interaction (i.e. lower value of k) results in a greater fraction of the case-control phenotype difference being attributable to epistasis. (PDF) [file pgen.1006003.s006.pdf]

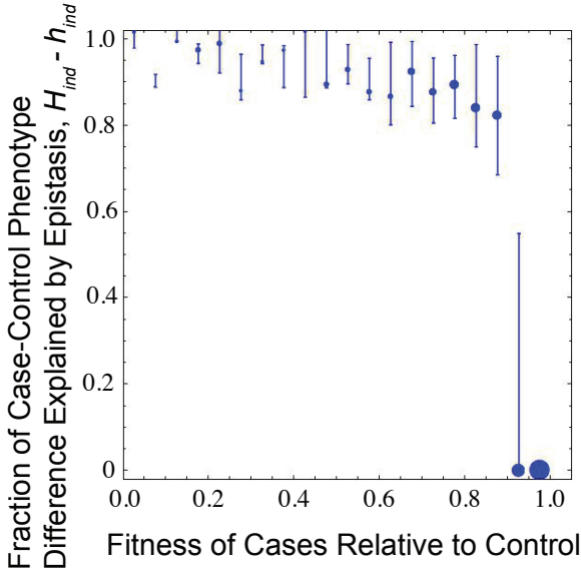

Supplement: S7 Fig — Summary of the data in Fig 6C. Circles indicate the median value of epistasis fraction, Hind—hind, for case individuals with similar fitness values, and error bars indicate the corresponding 25th and 75th percentile values. (PDF) [file pgen.1006003.s007.pdf]

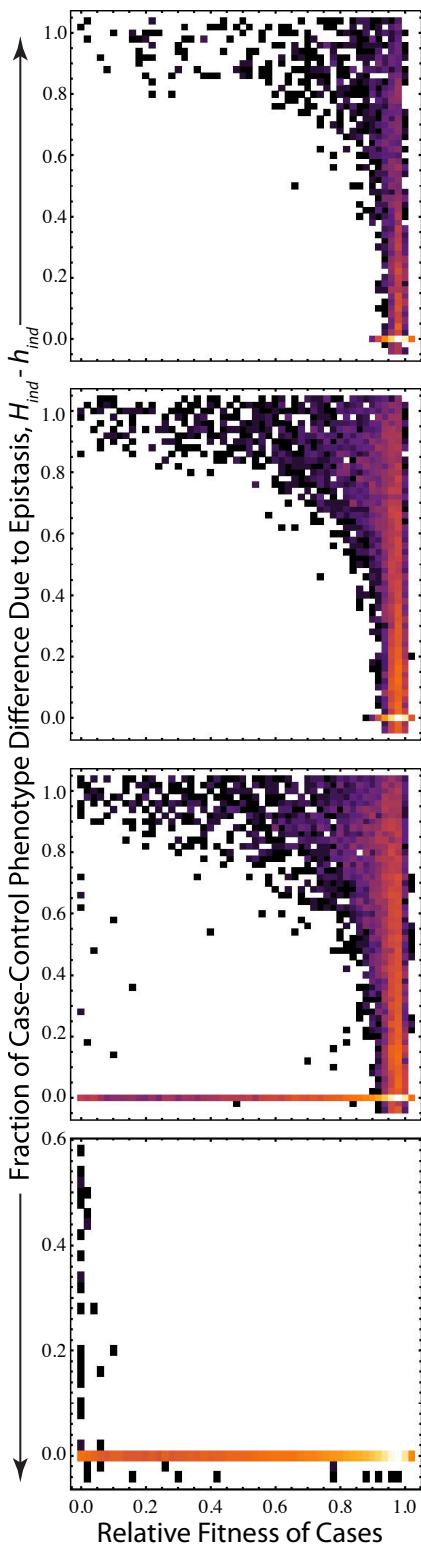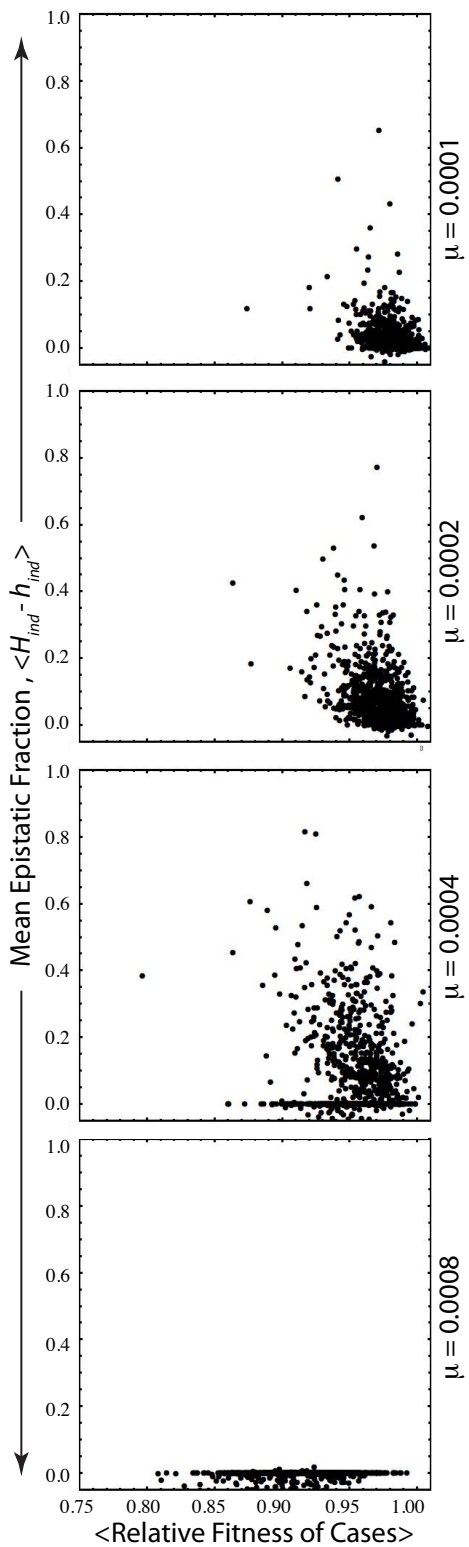

Supplement: S8 Fig — Mutation rates (per allele per generation) for each row are indicated on the right-hand side. The left-hand panels show the joint distribution of the epistatic fraction, Hind—hind, and fitness (relative to mean control fitness) over all cases and all sampled time points. In the right-hand panels, each dot represents a single time point at which the epistatic fraction and relative fitness were averaged over all cases. Parameter values are σdev = σenv = 0.05 and σμ = 1.0. (PDF) [file pgen.1006003.s008.pdf]

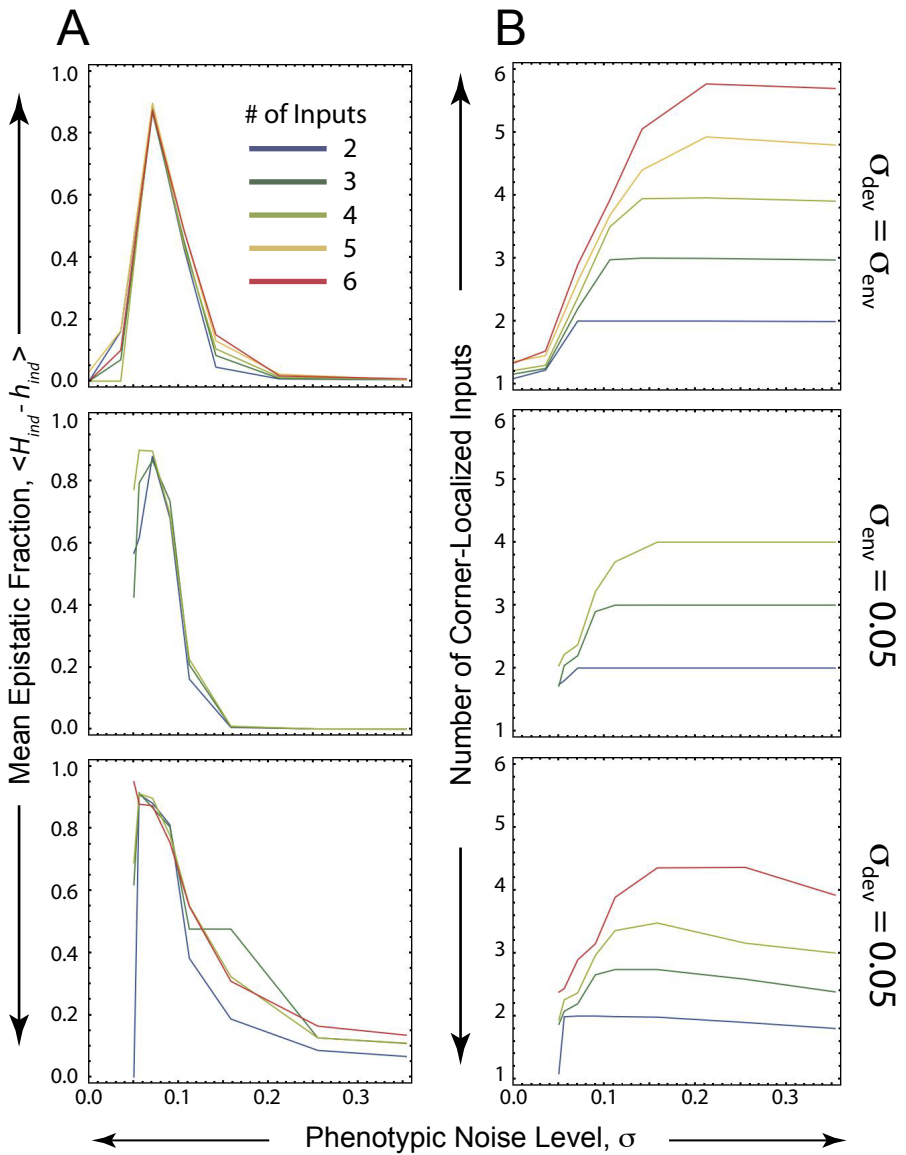

Supplement: S9 Fig — (A) Epistatic fraction Hind−hind averaged over all cases with a relative fitness of less than 0.9 and over all time points, as a function of the total phenotypic noise level σ=σdev2+​σenv2, illustrating the biphasic dependence of epistasis on noise level evident in Fig 6E and 6G. Epistasis decreases more with increasing developmental, versus environmental, noise (compare second and third rows). Increasing the dimensionality of the system has only a modest effect on the epistatic fraction, with the largest effect occurring when σenv > σdev. (B) The number of inputs that are localized to the corner, defined as the number of time- and population-averaged input values that are within one mutational step (σμ) of the corner, as a function of phenotypic noise level, σ=σdev2+​σenv2. Greater values of phenotypic noise are required to localize larger numbers of dimensions to the corner. As seen in Fig 5, increasing developmental noise monotonically enhances corner localization (second row) while intermediate levels of environmental noise correspond to maximum corner localization (third row). In both columns, rows differ in the relative contributions of σdev and σenv to σ. (PDF) [file pgen.1006003.s009.pdf]

$$\sigma_{\text{dev}} = \sigma_{\text{env}} = 0.05$$

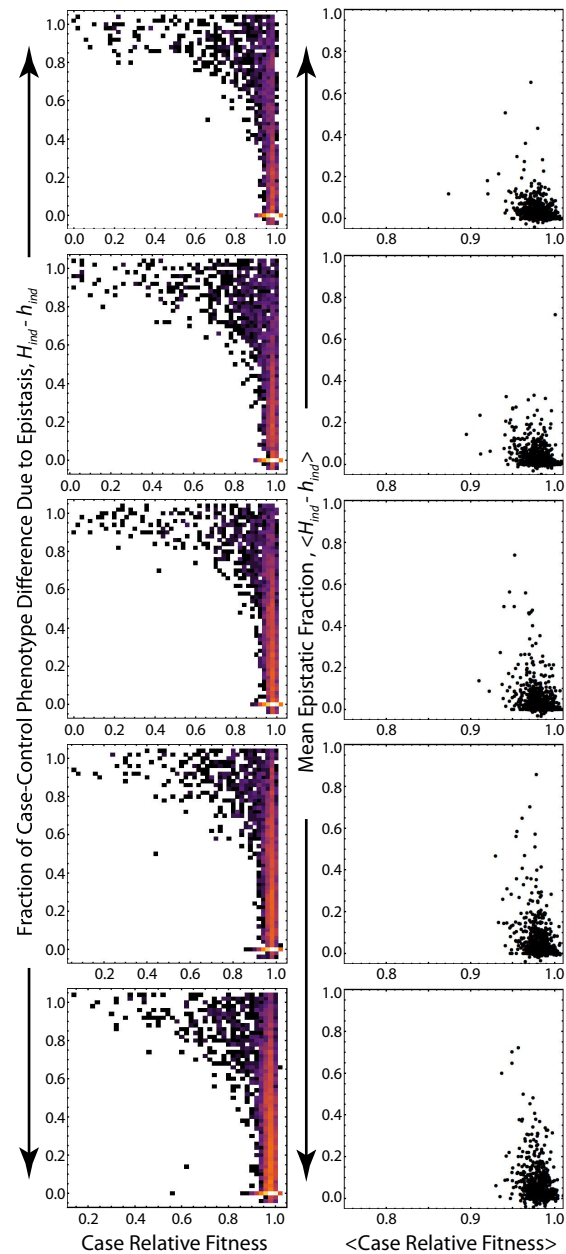

$$\sigma_{\text{dev}} = 0.05; \sigma_{\text{env}} = 0.15$$

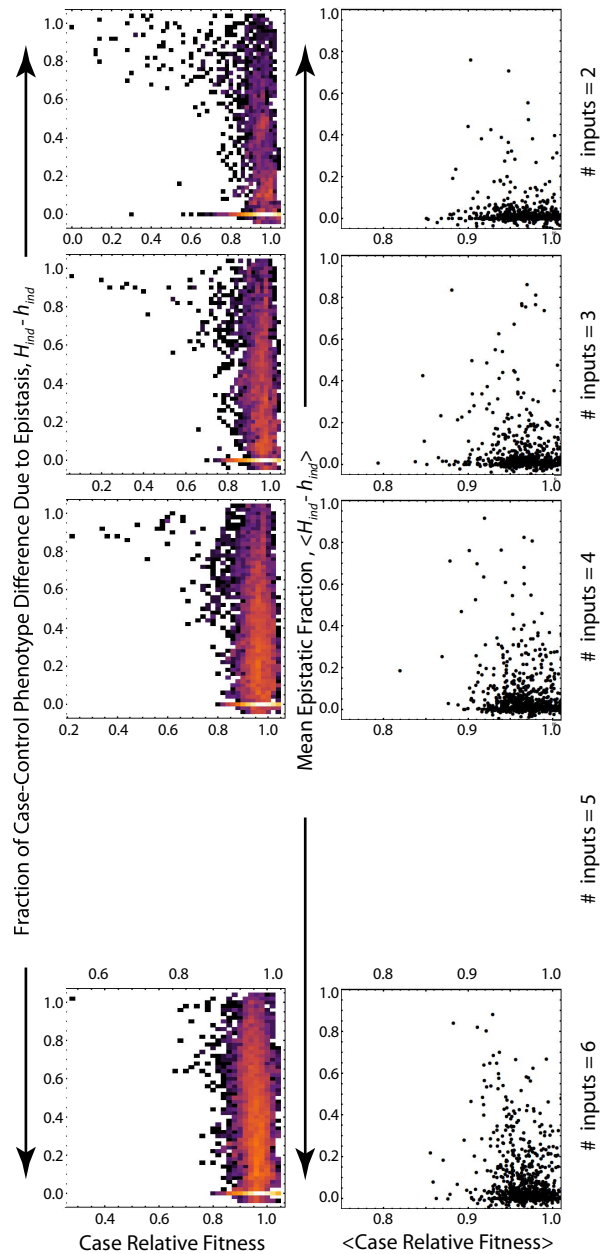

Supplement: S10 Fig — Each row corresponds to a different number of inputs in the LP model (indicated on the right-hand side). Each input value is determined by its own locus, and all loci are unlinked. The first and third columns show the joint distribution of the epistatic fraction, Hind—hind, and fitness (relative to mean control fitness) for all cases and all sampled time points. In the second and fourth columns, each dot represents a single time point, at which the mean values of the epistatic fraction Hind−hind and case relative fitness were calculated. The first and second column (σdev = σenv = 0.05) correspond to the condition under which we observed the strongest epistasis among cases with relative fitness < 0.9 (the peak in the first row of S9A Fig). The third and fourth column (σdev = 0.05; σenv = 0.15) correspond to the condition under which dimensionality appeared to have the greatest influence on the epistatic fraction for cases with relative fitness < 0.9 (σ = 0.158 in third row of S9A Fig). (PDF) [file pgen.1006003.s010.pdf]

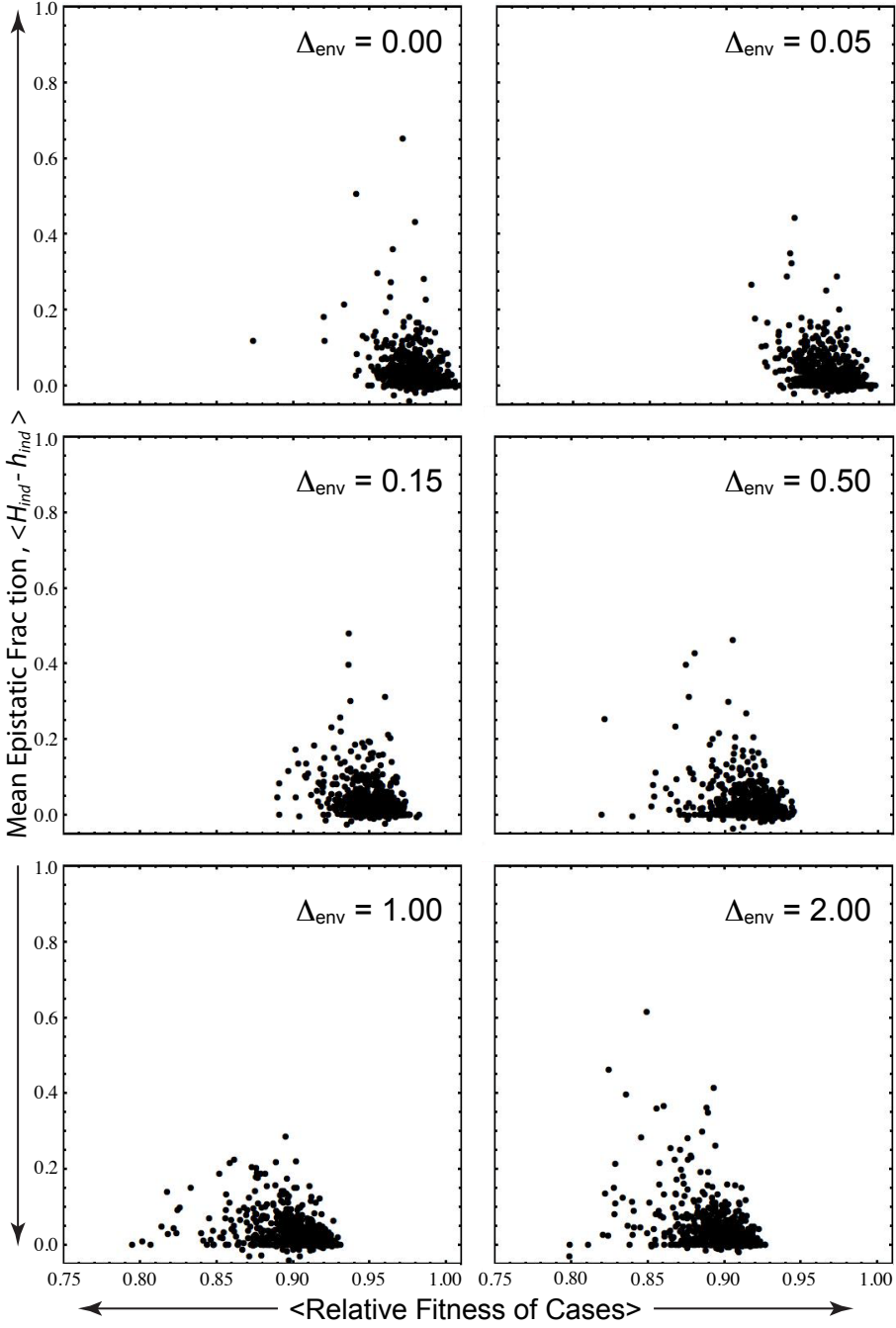

Supplement: S11 Fig — Simulations were run as described, but in this case an additional environmental shift was temporarily performed immediately prior to sampling case and control individuals at each time point (represented by points on the graphs). Environmental shifts were implemented by increasing both input values by a fixed quantity Δenv on a logarithmic scale (e.g., lnx became lnx + Δenv). Shifting the environment in this way reduces the fitness of cases relative to controls, but the reduction is limited by the fact that both cases and controls suffer a fitness cost at large environmental shifts. Parameter values were k = 0; s = 1; σdev = σenv = 0.05; σμ = 1; μ = 10−4. (PDF) [file pgen.1006003.s011.pdf]

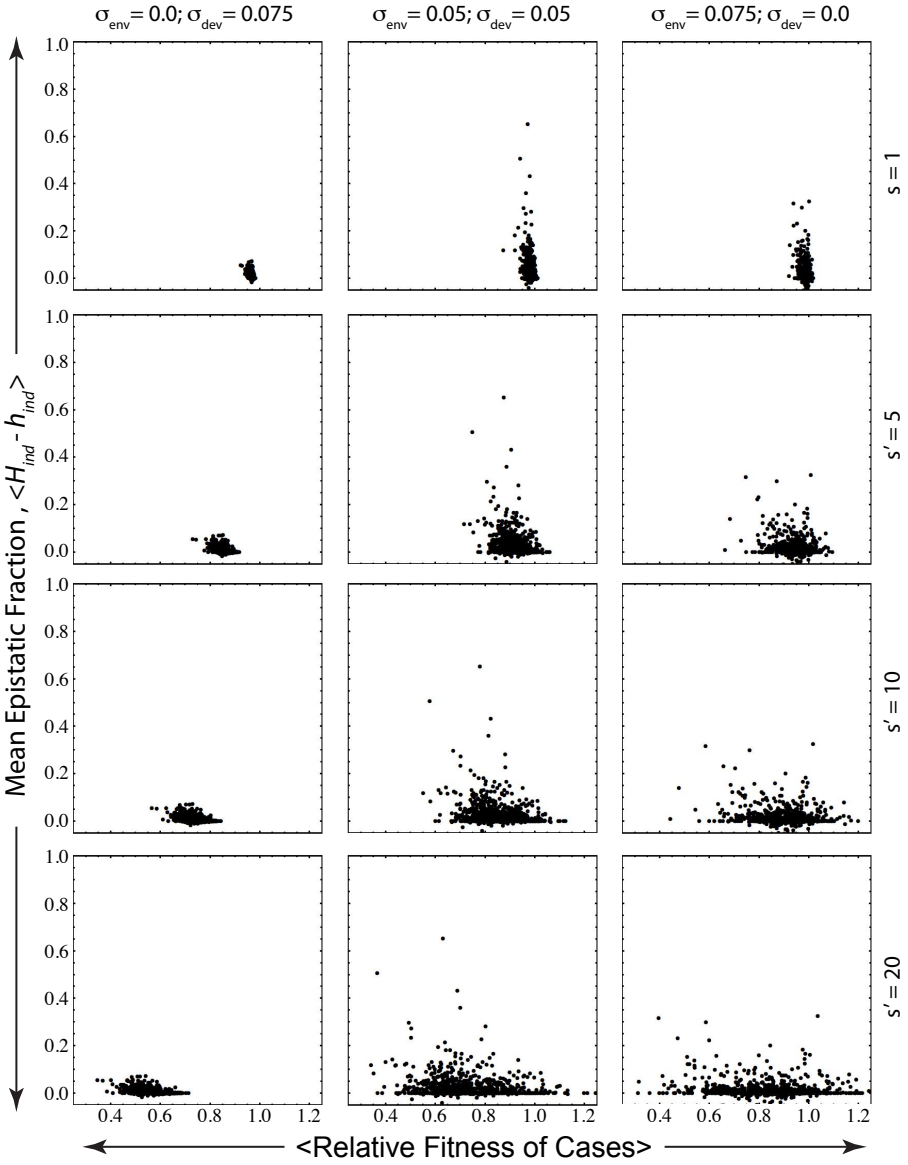

Supplement: S12 Fig — Simulations were run with selection strength s = 1 (top row). At each time point, we temporarily increased the strength of selection from s to s’ before averaging epistasis, Hind—hind, and relative fitness over all cases (second through third rows). Under pure developmental noise (left column, top row), all cases have reduced fitness relative to controls, reflecting the fact that the population is centered at the corner. In contrast, under pure environmental noise (right column, top row), values of case fitness extend upwards to 1 (and beyond), reflecting the fact that the population is not necessarily centered on the corner. That is, when environmental noise shifts the population so that most of its individuals have sub-optimal phenotype values, cases, which represent the individuals with the highest phenotype values, will have higher fitness than controls. Since increasing the strength of selection has little effect on high-fitness individuals, it can have a substantial impact on the relative fitness of cases and controls (second through third rows). Parameter values were k = 0 and σμ = 1. (PDF) [file pgen.1006003.s012.pdf]
