# Supplementary material for: Survival of the Curviest: Noise-Driven Selection for Synergistic Epistasis
Source: PLoS Genet. 2016 Apr 28;12(4):e1006003. doi: 10.1371/journal.pgen.1006003 (PMC4849581; doi:10.1371/journal.pgen.1006003)
Supplement: S2 Fig — (A-D) Distribution of input values among all individuals (green) in a simulated population and conditioned upon individuals being cases (pink) or controls (dark blue). Controls are drawn from the middle 50% of rank-ordered phenotype value (ranging from the 25th to the 75th percentile); cases represent the top 1% of this rank-ordered distribution. Since developmental noise is uncorrelated among individuals, the nominal input values of cases and controls are poorly differentiated (A), implying little heritability. Environmental noise, on the other hand, is correlated across individuals, resulting in a correlation between nominal input values and case-control status (C), and high heritability. (E-H) Time courses. Blue lines connect points corresponding to the average values of x and y in the population at intervals of 500,000 generations. Red bars indicate the magnitude of the variation in the population at each sampled timepoint (+/-2 standard deviations). (I-L) Median locations of case (pink) and control (dark blue) samples for the same temporal sequence of samples shown in panels E through H. Black lines connect control and case medians from the same time point. Parameter values are zopt = s = 1, N = 5000, k = 0.01, and σμ = σdev = σenv = 0.1 (PDF) [file pgen.1006003.s002.pdf]

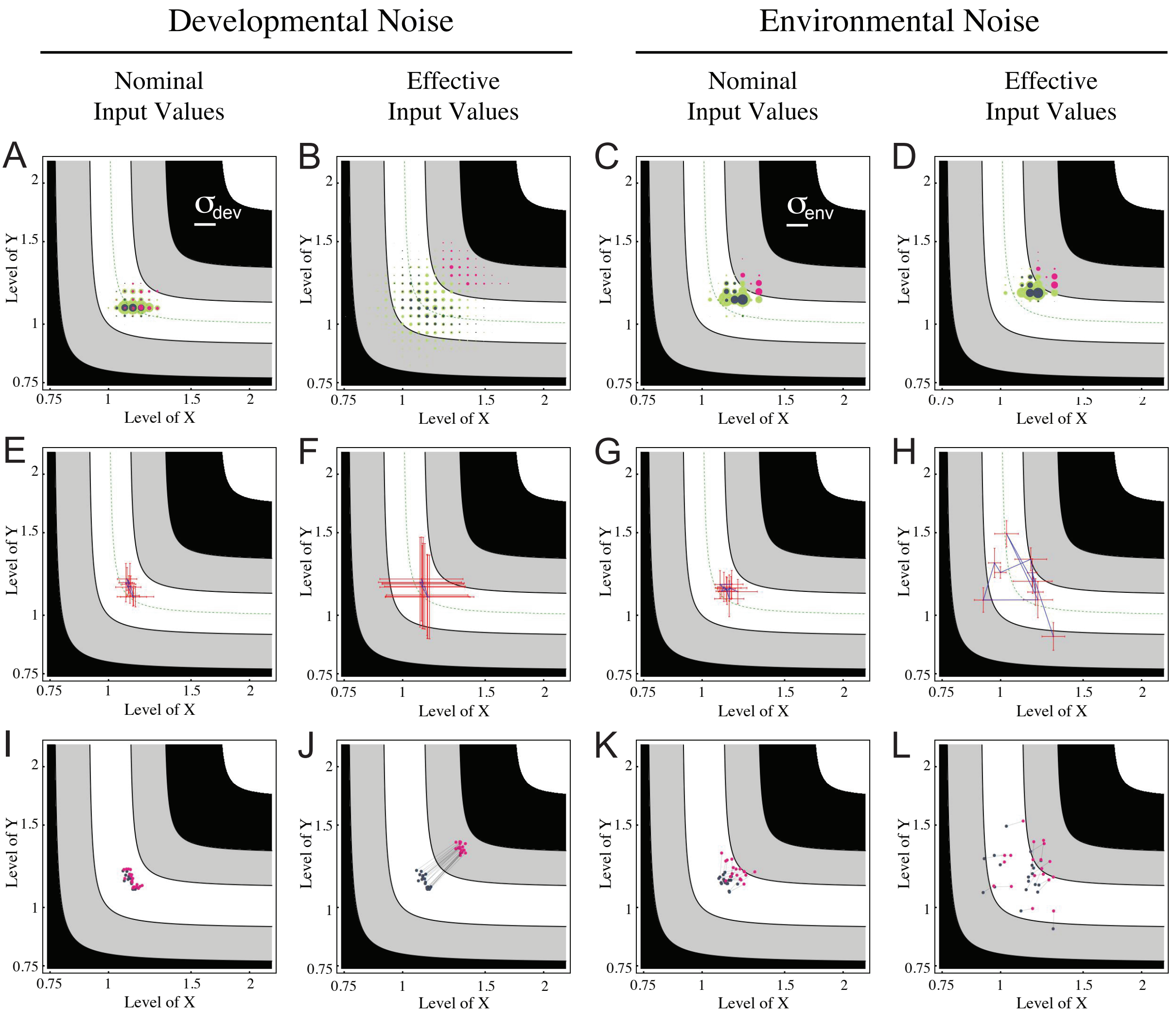

Developmental Noise
Environmental Noise
Nominal Input Values
Effective Input Values
Nominal Input Values
Effective Input Values
A
B
C
D
E
F
G
H
I
J
K
L
σdev
σenv
Level of X
Level of Y
0.75
1
1.5
2
