# Supplementary material for: Survival of the Curviest: Noise-Driven Selection for Synergistic Epistasis
Source: PLoS Genet. 2016 Apr 28;12(4):e1006003. doi: 10.1371/journal.pgen.1006003 (PMC4849581; doi:10.1371/journal.pgen.1006003)
Supplement: S4 Fig — Fraction of simulations in which the population-mean ratio of the two input values lay within the indicated bounds (1.2 for A and B, 1.4 for C and D, and 3.0 for E and F). Parameter values were zopt = s = 1 and N = 5000. At moderate noise levels (A, C, and E), environmental and developmental noise behave similarly in terms of localizing the population at the corner. At higher noise levels (B, D, and F), developmental noise localizes the population to the corner more effectively than does environmental noise. (PDF) [file pgen.1006003.s004.pdf]

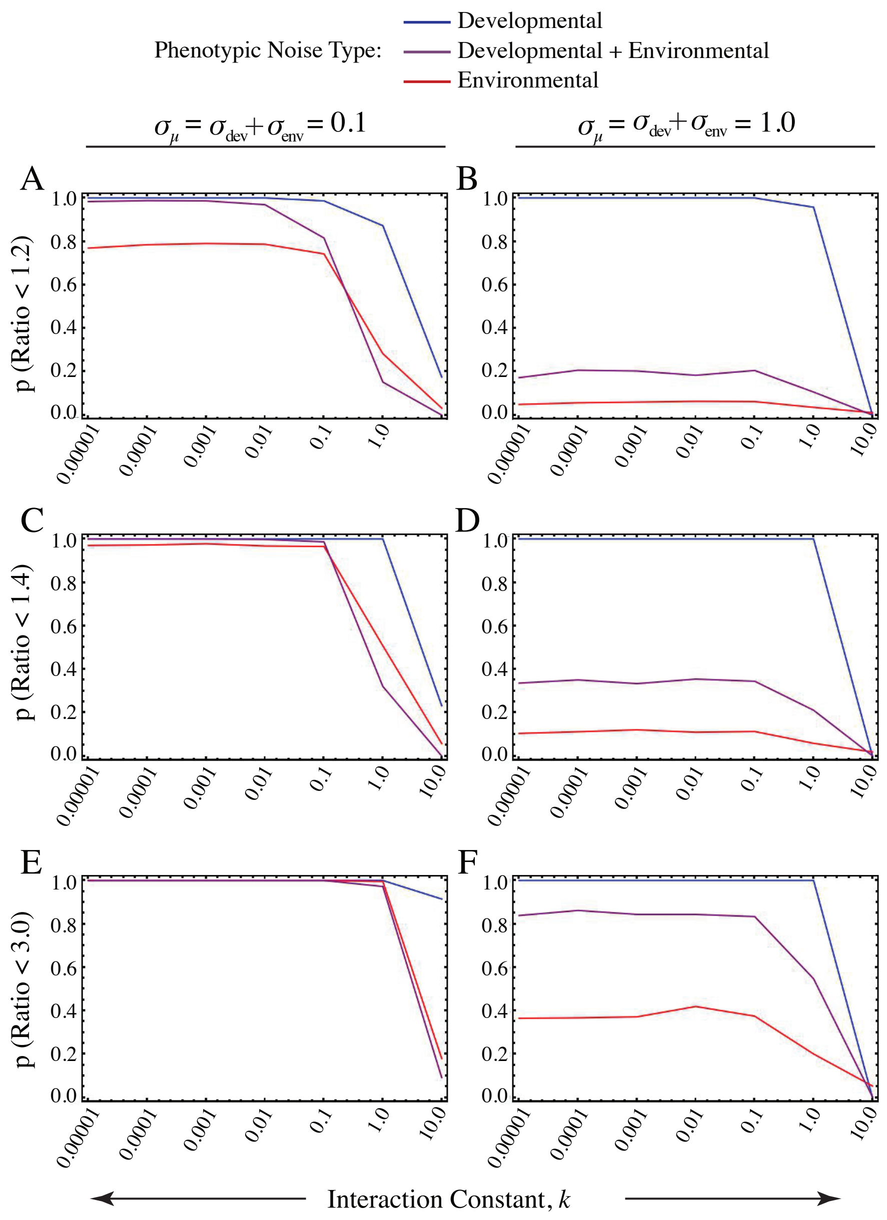

Phenotypic Noise Type:
Developmental
Developmental + Environmental
Environmental
$\sigma_\mu = \sigma_{dev} + \sigma_{env} = 0.1$
$\sigma_\mu = \sigma_{dev} + \sigma_{env} = 1.0$
A
B
C
D
E
F
p (Ratio < 1.2)
p (Ratio < 1.4)
p (Ratio < 3.0)
0.0
0.2
0.4
0.6
0.8
1.0
0.00001
0.0001
0.001
0.01
0.1
1.0
10.0
Interaction Constant, k
