# Supplementary material for: Survival of the Curviest: Noise-Driven Selection for Synergistic Epistasis
Source: PLoS Genet. 2016 Apr 28;12(4):e1006003. doi: 10.1371/journal.pgen.1006003 (PMC4849581; doi:10.1371/journal.pgen.1006003)
Supplement: S1 Text — (PDF) [file pgen.1006003.s013.pdf]

Text S1: Formulation and analysis of mathematical models for "Survival of the Curviest: Noise-Driven Selection for Synergistic Epistasis"

Jon F Wilkins, Peter T McHale, Joshua Gervin and Arthur D Lander

March 7, 2016

Contents

1 Epistasis at the individual versus population level **3**
1.1 Linear regression: general formulation 3
1.2 Linear regression: special case 4

2 Distribution of gene-values in case-control studies **6**

3 Random-walk approximation of evolution **7**
3.1 Evolution on the LP fitness landscape 7
3.2 Master equation of the random walk 7
3.3 Diffusion approximation 8
3.3.1 General stochastic process 8
3.3.2 Random walk 9
3.3.3 Moran model of fixation 10

4 Scaling argument for the LP model: $w(s, \sigma_i) = f(s\sigma_i^2)$ **11**

5 Distribution of gene-values in a population at mutation-selection balance **12**

6 Epistasis, orientation and curvature **13**
6.1 An expression for the maximum potential for functional epistasis at any location on a phenotypic landscape 13
6.2 The relationship between epistasis and orientation 15
6.3 The relationship between curvature and epistasis 16

7 Supporting References **18**

1 Epistasis at the individual versus population level

Here we derive the relationship shown in Fig S1C between epistasis at the individual level, which is dictated by the shape of the landscape where the individual resides, and population level, which is dictated by a planar fit to the landscape, weighted by the population distribution, i.e. linear regression.

1.1 Linear regression: general formulation

We begin by reviewing linear regression, e.g. (Lynch and Walsh, 1998; Ross, 2009; Neher and Shraiman, 2011). We perform the calculations in the asymptotic limit of a large sample size, i.e. we are not interested in stochastic variation in parameter estimates due to finite sample size.

Let $Z(\vec{x})$ describe the dependence of a quantitative trait on a set of genetically encoded quantitative input values $\vec{x} = (x, y)$, e.g., gene expression levels; for the sake of clarity of exposition we do not model environmental effects. From the function $Z(\vec{x})$ we can construct a random variable $Z(\vec{X})$ whose distribution depends on the probability density of gene values in the population $P(\vec{X} = \vec{x}) = p(\vec{x})$.

Narrow-sense heritability is

$$h^2_{pop*} = \frac{\sigma_A^2}{\sigma^2} \tag{S1}$$

where $\sigma^2 = \mathrm{var}(Z)$, σ_A^2 is the variance of a linear model of the true phenotypic landscape,

$$Z_A(\vec{x}) = \alpha + \beta_x x + \beta_y y, \tag{S2}$$

and the asterisk in h^2_{pop*} reminds us that we are not considering environmental effects. The coefficients of the linear model are determined by minimizing the population average of the random residual

$$\sigma_I^2 = \langle \Delta^2 \rangle = \int d\vec{x} p(\vec{x}) \Delta^2(\vec{x}), \tag{S3}$$

where the residual is defined by

$$\Delta(\vec{X}) = Z_A(\vec{X}) - Z(\vec{X}). \tag{S4}$$

Minimization with respect to α yields $\langle \Delta \rangle = 0$. This has two consequences. First, the optimal value of α is given by

$$\alpha = \langle Z \rangle - \beta_x \langle X \rangle - \beta_y \langle Y \rangle. \tag{S5}$$

Second, when α is optimal, we have the relation

$$\sigma_I^2 = \mathrm{var}(\Delta), \tag{S6}$$

implying that the least-squares optimization procedure minimizes the spread about the regression plane and therefore maximizes the amount of variation in Z that can be explained

by a linear combination of the input values. Minimization with respect to β_a yields

$$\beta_a = \frac{\chi_{az}\chi_{\bar{a}\bar{a}} - \chi_{a\bar{a}}\chi_{\bar{a}z}}{\chi_{xx}\chi_{yy} - \chi_{xy}^2}, \tag{S7}$$

where $(a, \bar{a}) = (x, y)$ or (y, x) and $\chi_{xy} = \text{cov}\,(X, Y)$, etc. It turns out that the residuals around the least-squares regression are uncorrelated with X and Y,

$$\text{cov}\,(X, \Delta) = \text{cov}\,(Y, \Delta) = 0. \tag{S8}$$

Notice, however, that the residual Δ may still depend on X, e.g. if Z is a nonlinear function of X. Using Eqs. (S6) and (S8), which are valid when the coefficients of the linear model are optimal, and the definition in Eq. (S4), the total variance can be decomposed as follows

$$\sigma^2 = \sigma_A^2 + \sigma_I^2. \tag{S9}$$

The variance not accounted for by the linear model, σ_I^2, is variously called "epistatic variance", "interaction variance" and "statistical epistasis", and is a measure of epistasis at the population level.

1.2 Linear regression: special case

Consider a genetic architecture defined by an inhomogeneous polynomial phenotype

$$Z(\vec{x}) = C\left[x + y + \frac{(xy)^n}{\xi^{2n-1}}\right] \tag{S10}$$

and an exponential gene-value distribution

$$p(\vec{x}) = \frac{1}{\lambda^2}e^{-(x+y)/\lambda}. \tag{S11}$$

The amplitude of the phenotypic landscape in Eq. (S10) is dictated by a constant C, while ξ represents the scale at which the landscape transitions from purely planer to purely synergistic, as reflected in the shape profile

$$h_{ind*}(x, x|0, 0) = \frac{2Z(x, 0)}{Z(x, x)} = \frac{2}{2 + (x/\xi)^{2n-1}}. \tag{S12}$$

Again, the asterisk reminds us that we are not considering environmental effects. (Notice that $h_{ind*} = h_{pop*}^2 = 1$ when Z is a plane.) According to the genotype distribution, Eq. (S11), the random variables X and Y are independent, which has been shown to be a reasonable assumption when h_{pop*}^2 is high (Neher and Shraiman, 2011). Using the "length" scale, λ,

defining the genotype distribution, we renormalize all variables as follows

$$\hat{\vec{x}} = \vec{x}/\lambda \tag{S13}$$

$$\hat{Z}(\hat{\vec{x}}) = Z(\vec{x})/\zeta \tag{S14}$$

$$\hat{p}(\hat{\vec{x}}) = \lambda^2 p(\vec{x}). \tag{S15}$$

where $\zeta = Z(\lambda, \lambda)$. Notice that the renormalized probability density is normalized,

$$\int d\hat{\vec{x}}\, \hat{p}(\hat{\vec{x}}) = 1, \tag{S16}$$

as it should be. Writing $\hat{h}_{ind*} = h_{ind*}(\lambda, \lambda|0, 0)$, we may cast the renormalized phenotype as

$$\hat{Z}(\hat{\vec{x}}) = \frac{1}{2}\hat{h}_{ind*}\,(\hat{x} + \hat{y}) + \left(1 - \hat{h}_{ind*}\right)(\hat{x}\hat{y})^n\,. \tag{S17}$$

The renormalized phenotype has the following properties

$$\hat{Z}(1, 0) = \hat{Z}(0, 1) = \frac{1}{2}\hat{h}_{ind*} \tag{S18}$$

$$\hat{Z}(1, 1) = 1. \tag{S19}$$

How is linear regression in the two spaces (i.e. bare versus renormalized variables) related? To approach that question, notice that the values of α and β_a $(a = x, y)$ that minimize σ_I^2 also minimize

$$\hat{\sigma}_I^2 = \sigma_I^2/\zeta^2 \tag{S20}$$

$$= \int d\hat{\vec{x}}\, \hat{p}(\hat{\vec{x}}) \left[\hat{Z}_A(\hat{\vec{x}}) - \hat{Z}(\hat{\vec{x}})\right]^2, \tag{S21}$$

where

$$\hat{Z}_A(\hat{\vec{x}}) = Z_A(\vec{x})/\zeta = \hat{\alpha} + \hat{\beta}_x\hat{x} + \hat{\beta}_y\hat{y} \tag{S22}$$

and

$$\hat{\alpha} = \alpha/\zeta \tag{S23}$$

$$\hat{\beta}_a = \beta_a\lambda/\zeta, \tag{S24}$$

which tells us how the regression coefficients in the two spaces are related. It follows that

$$\hat{\sigma}_A^2 = \mathrm{var}(\hat{Z}_A) = \sigma_A^2/\zeta^2. \tag{S25}$$

Using the relation $C = \frac{\zeta}{\lambda}\frac{1}{2}\hat{h}_{ind*}$ we may also show that

$$\hat{\sigma}^2 = \mathrm{var}(\hat{Z}) = \sigma^2/\zeta^2. \tag{S26}$$

It follows immediately that

$$\hat{h}^2_{pop*} = \frac{\hat{\sigma}^2_A}{\hat{\sigma}^2} = h^2_{pop*}. \tag{S27}$$

It follows that h^2_{pop*} depends on the parameters ξ and λ only through the shape of the landscape on the length scale defined by the population, $\hat{h}_{ind*}$, as confirmed by direct numerical calculations of h^2_{pop*} for a variety of values of ξ and λ (circles in Fig S1C; $\zeta = 1$; $n = 2$). Using the formulae in Section 1.1, one can show that the scaling curve is defined by

$$\hat{\sigma}^2_A = 2\left[\frac{1}{2}\hat{h}_{ind*} + (1-\hat{h}_{ind*})\,(n!)^2\,n\right]^2 \tag{S28}$$

$$\hat{\sigma}^2 = \frac{1}{2}\hat{h}^2_{ind*} + 2\hat{h}_{ind*}(1-\hat{h}_{ind*})\,(n!)^2\,n + (1-\hat{h}_{ind*})^2\left[((2n)!)^2 - (n!)^4\right], \tag{S29}$$

which is the line in Fig S1C.

2 Distribution of gene-values in case-control studies

In case-control studies, the proportion of the sample that are cases (i.e. $Z > z_t$, for some phenotype threshold z_t) is usually much larger than the prevalence in the population. Does the resulting enrichment for case genotypes, which are expected to have the greatest levels of functional epistasis, $1-h_{ind*}$, significantly increase statistical epistasis, $1-h^2_{pop*}$? To address this, we need the population distribution within the case-control sample, which is defined by

$$p_{cc}(\vec{x}) = P(\vec{X}_{cc} = \vec{x}) \tag{S30}$$

$$= (1-K_{cc})P(\vec{X} = \vec{x}|Z(\vec{X}) < z_t) + K_{cc}P(\vec{X} = \vec{x}|Z(\vec{X}) > z_t) \tag{S31}$$

where K_{cc} is the prevalence of disease in the case-control sample. The conditional probabilities can be written in terms of the disease prevalence in the population, $K = P(Z(\vec{X}) > z_t)$. For example,

$$P(\vec{X} = \vec{x}|Z(\vec{X}) > z_t) = \frac{P(\vec{X} = \vec{x}, Z(\vec{X}) > z_t)}{P(Z(\vec{X}) > z_t)} \tag{S32}$$

$$= \frac{p(\vec{x})}{K}\theta(Z(\vec{x}) > z_t), \tag{S33}$$

where θ is the unit step function. In summary,

$$p_{cc}(\vec{x}) = \frac{1-K_{cc}}{1-K}p(\vec{x})\theta(Z(\vec{x}) < z_t) + \frac{K_{cc}}{K}p(\vec{x})\theta(Z(\vec{x}) > z_t). \tag{S34}$$

Notice two things. First, $P(Z(\vec{X}_{cc}) > z_t) = K_{cc}$, as it should. Second, X_{cc} and Y_{cc} are not independent since $p_{cc}(x, y) \neq p_{cc}(x)p_{cc}(y)$.

3 Random-walk approximation of evolution

The evolution of a sufficiently small population can be mapped to a random walk. To see this, consider an initially monomorphic population, in which all N individuals have the same set of "input values" or "genotype". As a result of mutation (that occurs at rate μ per individual per gene per generation), the population becomes subdivided into sub-populations with different genotypes (i.e. it becomes polymorphic). When the population is small ($N\mu \leq 1$, as is the case in humans (Hartl and Clark, 2007, p165)), there are only a few such sub-populations, with each "drifting" in size until one "fixes" or "substitutes" while the others "extinguish". At that point, the population is again monomorphic, but with a new common genotype. We can thus approximate the population as a randomly walking "particle", which, upon arrival at a new position (genotype), builds up a "cloud" of minor-frequency polymorphisms before jumping to the next position (van Nimwegen et al., 1999; Poon and Otto, 2000; Berg et al., 2004; Sella and Hirsh, 2005; Bloom et al., 2006; Mustonen and Lässig, 2009; Serohijos et al., 2012).

3.1 Evolution on the LP fitness landscape

On the LP fitness landscape, strong selection quickly drives the particle (and its cloud) to a point of high fitness, after which it wanders slowly along a one-dimensional, relatively flat "ridge" (Fig. S3A). When gene interaction is weak, $k \gg 1$, the ridge is curved (Fig. S3A). In this regime, the particle "tacks": it moves along the ridge by alternate mutations in the x and y loci. Since this complication affects only the dynamics, and not the equilibrium, which is our primary concern, we did not explicitly model the weak interaction regime. When gene interaction is strong, $k \ll 1$, the ridge is comprised of two straight "arms", that intersect at a "corner". In this regime, mutations in one of the genes moves the particle along the corresponding arm (Fig. S3B).

The effect of phenotypic noise is to assign to the particle a fitness value randomly sampled from the particle's immediate vicinity. Provided sampling is much faster than the rate at which the particle jumps, we hypothesized that the particle's dynamics are governed by the space-averaged fitness. It turns out that, in the strong interaction regime, the profile of this "effective fitness" along the horizontal arm, $w_e(x)$, is graded, biasing the random walk of the particle towards the corner (Fig. S3C).

3.2 Master equation of the random walk

Here we describe mathematically how we modeled the random walk along the horizontal arm (strong interaction regime; $k \ll 1$). When the population-wide mutation rate is small, $N\mu \ll 1$, a new mutation likely extinguishes or fixes before the next mutation occurs, implying that the population consists of at most one mutant sub-population at any one time. In this regime, two things must happen for a particle, initially located at gene value x', to jump to gene value x. First, a mutation that alters the gene value in this way must occur in some individual; let $g(x|x')$ be the corresponding probability density that the value of the mutant allele is x, given that the gene's value prior to the mutation was x'. Second, the mutant individual must fix in the population, the probability of which we denote by $\rho(x|x')$.

Thus, like the Metropolis-Hastings algorithm in statistical physics, the particle jumps only if a move is both proposed (by mutation) and accepted (by fixation). Therefore, the probability density per mutation that the particle, initially at x', jumps to x is

$$\psi(x|x') = \delta(x - x')\,[1 - a(x')] + g(x|x')\rho(x|x'), \tag{S35}$$

where

$$a(x') = \int_{-\infty}^{\infty} g(s|x')\rho(s|x')ds. \tag{S36}$$

The coefficient of the delta function in Eq. (S35) is the probability that the proposed move is rejected. Representing mutations by a Poisson process with rate $N\mu$, the transition probability density per unit time (van Kampen, 2007, p96) is

$$W(x|x') = N\mu\psi(x|x'). \tag{S37}$$

The probability $P(x,t)$ that the particle is at x at time t is then governed by the master equation

$$\partial_t P(x,t) = \int_{-\infty}^{\infty} dx' \left\{W(x|x')P(x',t) - W(x'|x)P(x,t)\right\}. \tag{S38}$$

3.3 Diffusion approximation

The master equation, Eq. (S38), cannot be solved analytically. We must therefore resort to an approximation that is mathematically tractable. One such approximation is called the "diffusion approximation". Though excellent, the textbook presentation of this approximation (van Kampen, 2007, ch 11) contains flaws that hinder our use of it. Next, we present a correct derivation of the diffusion approximation of the master equation, Eq. (S38), suitable for our purposes.

3.3.1 General stochastic process

Our goal is to rewrite the master equation, Eq. (S38), as a power series expansion in a system-size parameter Ω. The parameter Ω creates a distinction between two scales. One scale is determined by the size of the jumps, $1/\Omega$, and will be denoted by the "extensive" variable X; the other scale is the one on which macroscopic properties of the system are measured and is indicated by the "intensive" variable $x = X/\Omega$. Following van Kampen, we write the transition probability density per unit time as

$$W(x|x') = f(\Omega)\left\{\Phi_0(x';R) + \frac{1}{\Omega}\Phi_1(x';R) + \cdots\right\}, \tag{S39}$$

where $R = X - X'$. Substituting this expression for $W(x|x')$ into the master equation, Eq. (S38), and changing the variable of integration from x' to R, we get

$$\begin{aligned}\partial_t P(x,t) = \frac{f(\Omega)}{\Omega}\int_{-\infty}^{\infty} dR \left\{ \Phi_0(x - \frac{R}{\Omega}; R) + \cdots \right\} P(x - \frac{R}{\Omega}, t) \\ -\frac{f(\Omega)}{\Omega}\int_{-\infty}^{\infty} dR \left\{ \Phi_0(x; -R) + \cdots \right\} P(x,t).\end{aligned} \tag{S40}$$

Taylor expanding the first integrand in the jump size, $1/\Omega$, we get

$$\begin{aligned}\partial_t P(x,t) = -\frac{f(\Omega)}{\Omega^2}\partial_x \left\{ \alpha_{10}(x) + \frac{1}{\Omega}\alpha_{11}(x) + \cdots \right\} P(x,t) \\ +\frac{1}{2}\frac{f(\Omega)}{\Omega^3}\partial_x^2 \left\{ \alpha_{20}(x) + \frac{1}{\Omega}\alpha_{21}(x) + \cdots \right\} P(x,t), \\ + \cdots,\end{aligned} \tag{S41}$$

where the "jump moments" are given by

$$\alpha_{\nu\lambda}(x) = \int_{-\infty}^{\infty} dR R^{\nu} \Phi_\lambda(x; R). \tag{S42}$$

3.3.2 Random walk

To apply the diffusion approximation to the random walk, we must cast its transition probability, Eq. (S37), in the power-series form of Eq. (S39). To this end, we write the distribution of mutational effects as

$$g(x|x') = \frac{1}{2}\frac{1}{\sigma_\mu} I_{-1,1}(R), \tag{S43}$$

where the *natural logarithm of* the difference in gene values, $x - x' = R\sigma_\mu$, is written in units of a mutation effect size, σ_μ (see Fig. S3D), and the indicator function $I_{a,b}(R)$ is defined to be 1 if $R \in [a, b]$ and 0 otherwise. On this scale, the delta function in Eq. (S35) is

$$\delta(x - x') = \frac{1}{\sigma_\mu}\delta(R). \tag{S44}$$

The fixation probability, considered as a function of the *natural logarithm of* gene value, x, is Taylor expanded about x'

$$\rho(x|x') = \rho(x'|x') + R\sigma_\mu\, \partial_s \rho(s|x')\big|_{s=x'} + \cdots \tag{S45}$$

Substituting Eqs. (S43) and (S45) into Eq. (S36) and changing the integration variable from s to $R = (s - x')/\sigma_\mu$, one gets

$$a(x') = \rho(x'|x') + \frac{1}{6}\sigma_\mu^2\, \partial_s^2 \rho(s|x')\big|_{s=x'} + \cdots \tag{S46}$$

Substituting Eqs. (S43), (S44), (S45) and (S46) into Eqs. (S35) and (S37), and comparing the resulting power series in σ_μ with the corresponding power series in $1/\Omega$, Eq. (S39), we infer that, for the random walk,

$$\begin{aligned} f &= N\mu/\sigma_\mu & \text{(S47)} \\ \Phi_0(x';R) &= \delta(R)\left(1-\rho(x'|x')\right)+\frac{1}{2}I_{-1,1}(R)\rho(x'|x') & \text{(S48)} \\ \Phi_1(x';R) &= \frac{1}{2}RI_{-1,1}(R)\,\partial_s\rho(s|x')|_{s=x'} & \text{(S49)} \end{aligned}$$

Substituting Eq. (S48) into Eq. (S42), we find that $\alpha_{10}=0$, which implies that the master equation of the random walk is of the "diffusion type" (van Kampen, 2007, ch 11) and the lowest order approximation arising from Eq. (S41) is a Fokker-Planck equation:

$$\partial_t P = f\sigma_\mu^3\left\{-\partial_x\alpha_{11}P+\frac{1}{2}\partial_x^2\alpha_{20}P\right\}. \quad \text{(S50)}$$

(Notice that this expression for the diffusion approximation differs by a factor of $\Omega=1/\sigma_\mu$ from the textbook version (van Kampen, 2007, Eqs. (1.5) and (1.6) on p274).) The prefactor on the right-hand side of the Fokker Planck equation is $f\sigma_\mu^3=N\mu\sigma_\mu^2$, while, under the Moran model of fixation (see Section 3.3.3), the jump moments are

$$\begin{aligned} \alpha_{11}(x) &= \frac{1}{3}\,\partial_s\rho(s|x)|_{s=x}=\frac{1}{6}\frac{N-1}{N}\frac{d\ln w_e(x)}{dx} & \text{(S51)} \\ \alpha_{20}(x) &= \frac{1}{3}\rho(x|x)=\frac{1}{3}\frac{1}{N}. & \text{(S52)} \end{aligned}$$

The Fokker Planck equation may therefore be written

$$\partial_\tau P(x,\tau)=-\partial_x\left[v(x)P(x,\tau)\right]+\partial_x^2P(x,\tau), \quad \text{(S53)}$$

where x is the natural logarithm of gene value, our new time variable is

$$\tau=\frac{1}{6}\mu\sigma_\mu^2 t, \quad \text{(S54)}$$

and the "velocity field" is

$$v(x)=(N-1)\frac{d\ln w_e(x)}{dx}. \quad \text{(S55)}$$

The steady-state solution is the Boltzmann distribution (Berg et al., 2004; Sella and Hirsh, 2005; Mustonen and Lässig, 2009)

$$P(x)\propto e^{(N-1)\ln w_e(x)}=w_e(x)^{N-1}. \quad \text{(S56)}$$

3.3.3 Moran model of fixation

Here we show how the jump moments in Eqs. (S51) and (S52) were calculated. Suppose that the population contains n mutant individuals, each with effective fitness w_e, and $N-n$ wild

type individuals, each with effective fitness w_e'. In any one time step, a random individual is chosen for reproduction in proportion to his fitness (and then replaced) and a random individual is chosen for elimination (without regard to fitness). The probabilities that, in the next time step, the number of mutants will be $n-1$, n or $n+1$ are

$$\begin{aligned} p_{n\to n-1} &= \frac{n}{N}\frac{w_e'(N-n)}{w_e n + w_e'(N-n)} && \text{(S57)}\\ p_{n\to n} &= 1 - p_{n\to n-1} + p_{n\to n+1} && \text{(S58)}\\ p_{n\to n+1} &= \frac{N-n}{N}\frac{w_e n}{w_e n + w_e'(N-n)} && \text{(S59)} \end{aligned}$$

respectively. This is a birth-death process, for which the probability of reaching the state where all individuals are mutant, conditioned on starting with just one mutant, is (Nowak, 2006, p99, Eq. (6.10))

$$\left\{1 + \sum_{j=1}^{N-1}\prod_{n=1}^{j}\frac{p_{n\to n-1}}{p_{n\to n+1}}\right\}^{-1} = \frac{1 - w_e'/w_e}{1-(w_e'/w_e)^N}, \tag{S60}$$

which approaches $1/N$ as $w_e \to w_e'$. Written as a function of the *natural logarithm of* gene value, x, and the effective fitness profile $w_e(x)$, the fixation probability is

$$\rho(s|x) = h(r(s,x)), \tag{S61}$$

where

$$\begin{aligned} h(r) &= \frac{1-r}{1-r^N} && \text{(S62)}\\ r(s,x) &= w_e(x)/w_e(s). && \text{(S63)} \end{aligned}$$

With regard to the "diffusion" jump moment, Eq. (S52), we immediately obtain (see Fig. S3E)

$$\rho(x|x) = 1/N. \tag{S64}$$

With regard to the "advection" jump moment, Eq. (S51), we find that

$$\begin{aligned} \left.\frac{\partial\rho(s|x)}{\partial s}\right|_{s=x} &= \left.\frac{dh}{dr}\right|_{r=1}\left.\frac{\partial r(s,x)}{\partial s}\right|_{s=x} && \text{(S65)}\\ &= \frac{1}{2}\frac{N-1}{N}\left.\frac{d\ln w_e(s)}{ds}\right|_{s=x}. && \text{(S66)} \end{aligned}$$

4 Scaling argument for the LP model: $w(s,\sigma_i) = f(s\sigma_i^2)$

Here, we show how the extended LP model depends on selection strength, s, and the level of noise, σ_i, through the product $s\sigma_i^2$, where by noise we mean either developmental ($i = \text{dev}$), environmental ($i = \text{env}$) or mutational ($i = \mu$). Eq. (2) of the main text shows that fitness,

w, depends only upon $s(\ln z - \ln z_{opt})^2$. To the extent to which the phenotype, z, is equal to one or the other gene level, x or y, the perturbation $(\ln z - \ln z_{opt})^2$ is either $(\ln x - \ln x_{opt})^2$ or $(\ln y - \ln y_{opt})^2$, each of which is a random variable with variance σ_i^2 (see Eq. (4) in main text). Hence, to a first approximation, w depends only on $s\sigma_i^2$.

5 Distribution of gene-values in a population at mutation-selection balance

We generalized the LP landscape to allow for a fitness ridge cross section that various along the optimal phenotype contour, both in terms of width and symmetry. On this generalized landscape, survival of the flattest can trump survival of the curviest, driving populations onto the arm and away from zones of high epistasis.

We wondered whether the shape of the population distribution of genes values could distinguish survival of the curviest and survival of the flattest. On an arm, the population distribution parallel to the arm is dictated mostly by mutation, which causes the population to wander en masse along the arm. Perpendicular to the arm, mutations are balanced by selection. We sought to model the resulting steady-state distribution of gene values in the direction orthogonal to the arm.

Let's model selection first. Let $\rho(x,t)dx$ be the number of individuals in a large population with allele value in the range $(x, x+dx)$ at time t. Selection can be modeled in discrete time,

$$\rho(x, t+1) = w(x)\rho(x,t), \tag{S67}$$

where $w(x)$ is the Wrightian fitness, or in continuous time,

$$\partial_t \rho(x,t) = m(x)\rho(x,t), \tag{S68}$$

where

$$m(x) = \ln w(x). \tag{S69}$$

is the Malthusian fitness. We shall adopt the continuous-time formulation. Let $p(x,t)$ denote the relative frequency of allele x at time t, i.e. $p(x,t)$ is the probability density

$$p(x,t) = \rho(x,t)/N(t), \tag{S70}$$

where

$$N(t) = \int_{-\infty}^{\infty} \rho(x,t)dx \tag{S71}$$

is the total number of individuals in the population at time t. Differentiating Eq. (S70) with respect to time and using the quotient rule yields

$$\partial_t p(x,t) = \left(m(x) - \overline{m}(t)\right) p(x,t), \tag{S72}$$

where $\overline{m}(t)$ is the population mean fitness defined by

$$\overline{m}(t) = \int_{-\infty}^{\infty} m(x)p(x,t)dx. \tag{S73}$$

.

Next, consider the effect of mutations. Let μ be the mutation rate per individual and $g(x|x')$ be the probability density that an individual changes its allelic value from x' to x upon mutation. Then the mutation influx to allele value x is

$$\partial_t p(x,t) = \int_{-\infty}^{\infty} g(x|x') \cdot \mu \cdot p(x',t)dx', \tag{S74}$$

while the mutation outflux is

$$\begin{aligned} \partial_t p(x,t) &= -\int_{-\infty}^{\infty} g(x'|x)dx' \cdot \mu \cdot p(x,t) & \text{(S75)} \\ &= -\mu p(x,t). & \text{(S76)} \end{aligned}$$

Adding Eqs. (S72, S74, S76) gives an integro-differential equation that governs the population distribution of gene values subject to the forces of selection and mutation,

$$\partial_t p(x,t) = (m(x) - \overline{m}(t))\, p(x,t) + \mu \left(\int_{-\infty}^{\infty} dx' g(x|x')p(x',t) - p(x,t) \right). \tag{S77}$$

This equation was first derived by Kimura to describe mutation-selection balance in the "continuum-of-alleles" model (Kimura, 1965). He investigated the steady-state solution under the assumptions that (1) fitness decreases quadratically from the optimum, $m(x) = -x^2$ (i.e. a Gaussian Wrightian fitness profile), and (2) production of new mutants follows a Gaussian density,

$$g(x|x') = \frac{1}{\sigma_\mu \sqrt{2\pi}}\, e^{-\frac{x^2}{2\sigma_\mu^2}}. \tag{S78}$$

With these assumptions, he approximated Eq. (S77) by a reaction-diffusion equation, yielding a Gaussian solution at steady state (Kimura, 1965). Note though that this is not the exact solution, which can differ significantly from normality under certain conditions (Burger, 1986).

6 Epistasis, orientation and curvature

6.1 An expression for the maximum potential for functional epistasis at any location on a phenotypic landscape

If we refer to the values associated with a pair of genes as x and y, and the phenotypic surface as $z = f(x,y)$, then for any pair of perturbations, δx and δy, to x and y, respectively, the

fraction of the phenotypic change explained by epistasis may be written as

$$1 - h_{\text{ind}*} = \frac{f(\delta\text{x} + x, \delta\text{y} + y) - f(\delta\text{x} + x, y) - f(x, \delta\text{y} + y) + f(x, y)}{f(\delta\text{x} + x, \delta\text{y} + y) - f(x, y)}.$$

Assuming δx and δy are small, we may use a Taylor Series expansion of f around x, y, or both together, to represent $f(x+\delta x, y)$, $f(x, y+\delta y)$ and $f(x+\delta x, y+\delta y)$ in terms of partial derivatives of f. In so doing, we simplify the above expression to

$$1 - h_{\text{ind}*} = \frac{\delta\text{x}\delta\text{y} f_{\text{xy}} + \cdots}{\delta\text{x} f_x + \delta\text{y} f_y + \delta\text{x}\delta\text{y} f_{\text{xy}} + \cdots}, \tag{S79}$$

where "$\cdots$" refers to the higher order terms of the expansion.

We want to obtain a measure of epistasis that is not a function of the particular choices of δx and δy, but rather characterizes the general behavior at a particular location on a phenotypic landscape. One way to do this is to divide the above expression by the "average" mutational size, and then find the limiting behavior as this size goes to zero. In effect, we are asking how much epistasis is associated with a pair of infinitesimally small perturbations, relative to the average perturbation size. There are a number of ways we could represent the "average" perturbation size, but here we will use a root-mean-square of δx and δy, which we will call δ:

$$\delta = \sqrt{\frac{\delta\text{x}^2 + \delta\text{y}^2}{2}}. \tag{S80}$$

We will introduce a new parameter,

$$\varphi = \frac{\delta y}{\delta x}, \tag{S81}$$

which quantifies the relative magnitudes of the x and y perturbations. If perturbations to x and y go in the same direction, φ is positive; if not, φ is negative. If perturbations are of the same absolute magnitude, $|\varphi| = 1$. Dividing $1 - h_{ind*}$ by δ and taking the limit as $\delta \to 0$ (upon which all of the higher order terms in the Taylor series expansion vanish), gives

$$\lim_{\delta\to\infty} \frac{1 - h_{\text{ind}*}}{\delta} = \pm \frac{\sqrt{2}\varphi f_{\text{xy}}}{\sqrt{1+\varphi^2}\,(f_x + \varphi f_y)}. \tag{S82}$$

Since we are only interested in the absolute value of this quantity, which we shall call $\mathcal{E}$, the sign information here is irrelevant, and thus we have

$$\mathcal{E} = \left| \frac{\sqrt{2} f_{\text{xy}}}{\sqrt{1+\varphi^2}\left(\frac{1}{\varphi} f_x + f_y\right)} \right|. \tag{S83}$$

Now in considering any given system, we note that the units in which x and y are expressed are arbitrary. We may always choose those units so that the absolute magnitudes of perturbations in x and y are equal; we simply need to express f in terms of the same units. Then φ equals either 1 or -1. Thus, letting F represent the function f defined in

terms of the appropriate units of x and y, we get that epistasis, $\mathcal{E}$, can be either of

$$\left|\frac{F_{\mathrm{xy}}}{F_y + F_x}\right| , \left|\frac{F_{\mathrm{xy}}}{F_y - F_x}\right| \tag{S84}$$

depending upon the direction of the perturbations relative to each other. It can be shown that the maximum of these two expressions is always equal to

$$\mathcal{E}_{\mathrm{max}} = \left|\frac{F_{\mathrm{xy}}}{|F_y| - |F_x|]}\right| . \tag{S85}$$

That is, $\mathcal{E}_{\mathrm{max}}(x, y)$ is the maximum possible epistasis at the location (x, y).

On the other hand, in many biologically relevant scenarios, we are primarily concerned with landscapes on which we consider only perturbations to x and y that produce changes in the same direction, i.e. $f_x \delta x$ has the same sign as $f_y \delta y$, hence $\frac{1}{\varphi} f_x$ has the same sign as f_y. Under these conditions,

$$\left|\frac{1}{\varphi} f_x + f_y\right| = \left|\frac{1}{\varphi}\right| |f_x| + |f_y| , \tag{S86}$$

and Eq. (S83) reduces to

$$\mathcal{E}_* = \left|\frac{F_{\mathrm{xy}}}{|F_y| + |F_x|}\right| , \tag{S87}$$

where again F represents the function f defined in terms of the appropriately chosen units of x and y. Note that it is always the case that

$$\mathcal{E}_{\mathrm{max}} > \mathcal{E}_* \geq 0. \tag{S88}$$

As we shall see below, it is also useful to have a third definition of functional epistasis,

$$\hat{\mathcal{E}}_* = \frac{|F_{\mathrm{xy}}|}{\sqrt{{F_y}^2 + {F_x}^2}} , \tag{S89}$$

which is simply the absolute value of the mixed derivative of F normalized to the magnitude of the gradient of F. Note that since

$$|a| + |b| < \sqrt{a^2 + b^2} < \sqrt{2}(|a| + |b|) \; \forall \; a \text{ and } b, \tag{S90}$$

$\hat{\mathcal{E}}_*$ is bounded below by $\frac{1}{\sqrt{2}}\mathcal{E}_*$ and above by $\mathcal{E}_*$. Thus $\hat{\mathcal{E}}_*$ is a reasonably good estimator of $\mathcal{E}_*$, and the more different the absolute values of F_x and F_y, the better an estimator it is.

6.2 The relationship between epistasis and orientation

For a phenotypic surface $z = F(x,y)$, then the Optimal Fitness Countour (OFC) is the x-y curve defined by $F(x,y) = z_{\mathrm{opt}}$, where z_{opt} is a constant representing the "optimal" phenotype. The slope of the OFC may be related to the partial derivatives of the phenotypic surface F at the (x,y) points that lie along the OFC, according to the relationship $y'(x) = -F_x/F_y$.

Accordingly, the notion of the "orientation" of the OFC, as described in the main text, is equivalent to a measure of the relative magnitudes of F_x and F_y. A diagonal orientation means $|F_x| \approx |F_y|$. Horizontal and vertical orientation correspond to $|F_y| \gg |F_x|$ and $|F_y| \ll |F_x|$, respectively.

For landscapes in which "fall-off" does not vary significantly as one moves small distances along the OFC (i.e. fall-off is relatively uniform, as in Fig. 7B), epistasis at corners is expected to be zero for horizontal and vertical orientations but non-zero for a diagonal orientation: When the orientation of the corner is vertical, small changes to y produce no change in F, i.e. $F_y = 0$ at the corner. Having fall-off be uniform, i.e. locally independent of distance along the contour, implies that neighboring phenotype contours are locally parallel to the corner under consideration. A consequence of this is that F_y is invariant to changes in x, i.e. $F_{yx} = F_{xy} = 0$. A similar argument can be made for horizontally oriented corners using F_x to arrive at the identical conclusion. In contrast, at corners that are not horizontal or vertical, on landscapes with uniform fall-off, F_y will generally depend on x and F_x will depend on y, so that, in general, F_{xy} will be non-zero. Since F_{xy} serves as the numerator for both $\mathcal{E}_{max}$ and $\mathcal{E}_*$, we conclude that $\mathcal{E}_{max} = \mathcal{E}_* = 0$ at horizontally or vertically oriented corners, whereas these measures of epistasis are generally non-zero at diagonally oriented corners.

It is also apparent that, for exactly diagonal orientation, $\mathcal{E}_{\max} \to \infty$, whereas $\mathcal{E}_*$ does not. This is a consequence of our defining epistasis as the ratio between the phenotypic change when x and y are both perturbed to the phenotypic change predicted by the sum of the individual effects of perturbing x and y. If those individual perturbations happen to have exactly opposite effects, the phenotypic change predicted by their sum will be zero, so any phenotypic effect of perturbing them together will correspond to infinite epistasis. This is one reason why, as discussed above, $\mathcal{E}_*$ may sometimes be more biologically useful than $\mathcal{E}_{\max}$, even though the former only applies to a subset of the cases of gene-gene interaction included in the latter. It should also be noted that, as orientation moves away from the diagonal $\mathcal{E}_{\max}$ and $\mathcal{E}_*$ approach each other.

6.3 The relationship between curvature and epistasis

We've noticed empirically that locations on the phenotypic landscape where the OFC is highly curved are often associated with high epistasis. For any curve in the x-y plane that is expressed parametrically as $(x(t), y(t))$, the curvature $\mathcal{C}$ (i.e. the inverse of the radius of curvature) may be written as

$$\mathcal{C}(t) = \left| \frac{x'(t)y''(t) - y'(t)x''(t)}{(x'(t)^2 + y'(t)^2)^{3/2}} \right|. \tag{S91}$$

By definition, along the OFC, $F(x(t), y(t)) = z_{opt}$ for all t, so the derivative of F with respect to t must be zero. Taking the derivative with respect to t, gives us

$$y'(t)F_y + x'(t)F_x = 0. \tag{S92}$$

Hence (as long as $F_y \neq 0$), $y'(t) = \frac{-x'[t]F_x}{F_y}$. (If $F_y = 0$, then x'$(t) = \frac{-y'[t]F_y}{F_x}$, and if both F_x and F_y are zero, then $\mathcal{C}$ is undefined). Next, taking the derivative of Eq. (S92) with respect

to t gives us:

$$y''(t) = \frac{x'(t)\left(-F_y\left(F_{xx}x'(t)+F_{xy}y'(t)\right)+F_x\left(F_{xy}x'(t)+F_{yy}y'(t)\right)\right)-F_xF_yx''(t)}{F_y^2}. \tag{S93}$$

These expressions allow us to re-define $\mathcal{C}$ in terms of derivatives of F with respect to x and y:

$$\mathcal{C} = \frac{\left|F_{\rm yy}{F_x}^2-2F_xF_yF_{\rm xy}+{F_y}^2F_{\rm xx}\right|}{\left({F_y}^2+{F_x}^2\right)^{3/2}} \tag{S94}$$

$$= \left|\frac{-2F_xF_y}{{F_y}^2+{F_x}^2}\frac{F_{\rm xy}}{\sqrt{{F_y}^2+{F_x}^2}}+\frac{{F_x}^2}{{F_y}^2+{F_x}^2}\frac{F_{\rm yy}}{\sqrt{{F_y}^2+{F_x}^2}}+\frac{{F_y}^2}{{F_y}^2+{F_x}^2}\frac{F_{\rm xx}}{\sqrt{{F_y}^2+{F_x}^2}}\right| \tag{S95}$$

We notice that $\mathcal{C}$ can be viewed as being the sum of three terms $\frac{F_{\rm xy}}{\sqrt{{F_y}^2+{F_x}^2}}$, $\frac{F_{\rm yy}}{\sqrt{{F_y}^2+{F_x}^2}}$, and $\frac{F_{\rm xx}}{\sqrt{{F_y}^2+{F_x}^2}}$, weighted by three pre-factors $\frac{-2F_xF_y}{{F_y}^2+{F_x}^2}$, $\frac{{F_x}^2}{{F_y}^2+{F_x}^2}$ and $\frac{{F_y}^2}{{F_y}^2+{F_x}^2}$. The first of the three terms is simply a signed version of $\hat{\mathcal{E}}_*$. The second two represent the second derivatives of F with respect to x, and y, each normalized to the magnitude of the gradient of F. We shall rename them $\mathcal{F}_{\rm yy}$ and $\mathcal{F}_{\rm xx}$ to denote the fact that they, like $\hat{\mathcal{E}}_*$, can be viewed as a scaled measure of one of the second-order derivatives of F. The prefactors are most conveniently expressed as functions of the angle θ, formed between the tangent line to the OFC and the x-axis, i.e. $y'(x) = -F_x/F_y = \tan\theta$. Making this substitution, and taking note of the possible signs of the signed version of $\hat{\mathcal{E}}_*$, we get

$$\mathcal{C} = \left|2\sin\theta\cos\theta\hat{\mathcal{E}}_* \pm \left(\sin^2\theta\mathcal{F}_{\rm yy} + \cos^2\theta\mathcal{F}_{\rm xx}\right)\right|. \tag{S96}$$

This expression tells us that epistasis and curvature will correlate strongly with each other whenever the term in the parentheses is small compared with the term before it. For this to happen, two things must be the case.

First, θ cannot be too close to 0, $\frac{\pi}{2}$, or π, i.e. the orientation of the OFC cannot be horizontal or vertical. As we have already shown above, such orientations also minimize epistasis. If the OFC has the optimal orientation, i.e. diagonal, then the above expression reduces to

$$\mathcal{C} = \left|\hat{\mathcal{E}}_* \pm \frac{1}{2}\left(\mathcal{F}_{\rm yy} + \mathcal{F}_{\rm xx}\right)\right|. \tag{S97}$$

From this we see that, in order for epistasis and curvature to correlate strongly, epistasis should be larger than the average of $\mathcal{F}_{\rm yy}$ and $\mathcal{F}_{\rm xx}$. When both things are the case (favorable orientation and $\hat{\mathcal{E}}_* \gg \left|\frac{1}{2}\left(\mathcal{F}_{\rm yy} + \mathcal{F}_{\rm xx}\right)\right|$), curvature and epistasis will tend to correlate strongly with each other.

In the most general sense, we may interpret these two conditions as statements about how variation at genes x and y contributes to phenotype F. The first condition tells us that, in order for epistasis to be strong, F should be similarly sensitive to both x and y (as opposed

to being much more sensitive to one than the other). The second condition tells us that curvature will be a good predictor of epistasis whenever the nonlinearity of the phenotypic landscape derives more from the interaction between x and y, than from the independent effects of x and y themselves.

7 Supporting References

Johannes Berg, Stana Willmann, and Michael Lässig. Adaptive evolution of transcription factor binding sites. *BMC Evolutionary Biology*, 4(1):42, 2004.

J D Bloom, A Raval, and C O Wilke. Thermodynamics of Neutral Protein Evolution. *Genetics*, 175(1):255–266, October 2006.

R Burger. On the maintenance of genetic variation: global analysis of Kimura's continuum-of-alleles model. *Journal of mathematical biology*, 24(3):341–351, 1986.

Daniel L Hartl and Andrew G Clark. *Principles of Population Genetics*. Sinauer Associates Inc., 4th edition, 2007.

M Kimura. A stochastic model concerning the maintenance of genetic variability in quantitative characters. *Proceedings of the National Academy of Sciences of the United States of America*, 54(3):731–736, September 1965.

M Lynch and B Walsh. *Genetics and Analysis of Quantitative Traits*. Sinauer, 1998.

Ville Mustonen and Michael Lässig. From fitness landscapes to seascapes: non-equilibrium dynamics of selection and adaptation. *Trends in genetics*, 25(3):111–119, March 2009.

Richard A Neher and Boris I Shraiman. Statistical genetics and evolution of quantitative traits. *Reviews of Modern Physics*, 83(4):1283–1300, January 2011.

M A Nowak. *Evolutionary Dynamics: Exploring the Equations of Life*. Belknap Press (Harvard University Press), 1st edition, 2006.

A Poon and S P Otto. Compensating for our load of mutations: freezing the meltdown of small populations. *Evolution; international journal of organic evolution*, 54(5):1467–1479, October 2000.

Sheldon M Ross. Chapter 9 - REGRESSION. In Sheldon M Ross, editor, *Introduction to Probability and Statistics for Engineers and Scientists (Fourth Edition)*, pages 353–439. Academic Press, Boston, 2009.

G Sella and A E Hirsh. The application of statistical physics to evolutionary biology. *Proceedings of the National Academy of Sciences*, 102(27):9541–9546, July 2005.

Adrian W R Serohijos, Zilvinas Rimas, and Eugene I Shakhnovich. Protein Biophysics Explains Why Highly Abundant Proteins Evolve Slowly. *Cell Reports*, 2(2):249–256, August 2012.

N G van Kampen. *Stochastic Processes in Physics and Chemistry.* Elsevier, 3rd edition, 2007.

E van Nimwegen, J P Crutchfield, and M Huynen. Neutral evolution of mutational robustness. *Proceedings of the National Academy of Sciences*, 96(17):9716–9720, August 1999.
